# Unraveling the Hierarchical Self‐Assembly of Amphiphilic Block Copolymer‐Peptide Conjugates by Tip‐Enhanced Raman Spectroscopy

**DOI:** 10.1002/smll.202502157

**Published:** 2025-06-16

**Authors:** Christiane Höppener, Fabian H. Sobotta, Stephanie Hoeppener, Volker Deckert, Johannes C. Brendel

**Affiliations:** ^1^ Leibniz Institute of Photonic Technology (IPHT) Albert‐Einstein‐Straße 9 D‐07745 Jena Germany; ^2^ Institute of Physical Chemistry (IPC) and Abbe Center of Photonics Friedrich Schiller University Jena Helmholtzweg 4 07743 Jena Germany; ^3^ Jena Center for Soft Matter (JCSM) Friedrich Schiller University Jena Philosophenweg 7 07743 Jena Germany; ^4^ Laboratory of Organic and Macromolecular Chemistry (IOMC) Friedrich Schiller University Jena Humboldtstrasse 10 07743 Jena Germany; ^5^ Macromolecular Chemistry I University of Bayreuth Universitätsstr. 30 95447 Bayreuth Germany; ^6^ Bayreuth Institute of Macromolecular Research (BIMF) and Bavarian Polymer Institute (BPI) University of Bayreuth Universitätsstr. 30 95447 Bayreuth Germany; ^7^ Present address: Institute for Complex Molecular Systems Department of Chemical Engineering and Chemistry Eindhoven University of Technology P.O. Box 513 5600 MB Eindhoven Netherlands

**Keywords:** beta‐sheet, hierarchical structures, hydrogen bonds, persistence length, TERS spectroscopy

## Abstract

Self‐assembly of block copolymers in solution provides access to different nanostructures depending on block composition and processing conditions. However, more complex hierarchical nanostructures as found in nature remain challenging to achieve. In this study, the influence of a β‐sheet forming tetrapeptide sequence (GFFG) is investigated at the interface of an amphiphilic block copolymer based on poly(butyl acrylate) (PBA) and poly(ethylene oxide) (PEO). Using atomic force microscopy (AFM) and tip‐enhanced Raman spectroscopy (TERS), nanoscale insights are provided into the structural organisation and mechanical properties of these hybrid materials. Both the tetrapeptide‐containing block copolymer and a control block copolymer without the peptide linker form wormlike micelles in water. However, the incorporation of the peptide linker alters the micelle morphology by increasing the contour length sixfold compared to the control polymer and by altering the mechanical properties of the wormlike micelles. TERS analysis confirms the presence of ordered *β*‐sheet structures at the hydrophilic/hydrophobic interface, which increase the bending stiffness of the micelles. The introduction of additional secondary interactions, such as those induced by the peptide linker, therefore appears as an interesting lever to manipulate the structure formation and mechanical properties block copolymer micelles, opening up interesting design strategies for tailor‐made hierarchically structured nanomaterials.

## Graphical abstract

This study investigates peptide‐functionalized block copolymers forming wormlike micelles. Atomic force microscopy (AFM) and tip‐enhanced Raman spectroscopy (TERS) reveal that β‐sheet structures at the polymer interface enhance hierarchical organization. The peptide linker increases micelle contour length and bending stiffness. These findings suggest that introducing secondary interactions offers a promising strategy for designing advanced, hierarchically structured nanomaterials.

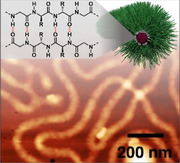

## 1 Introduction

The self‐assembly and organization of natural macromolecules into hierarchically ordered nanostructures comprising different shapes and sizes represent a process, which is omnipresent in nature. In synthetic materials, the creation of 3D nanostructures with defined topology still represents a highly challenging objective.^[^ ^1^ ^]^ In this regard, the self‐assembly of block copolymers in selective solvents has certainly attracted most attention to obtain core‐shell nanostructures of various morphologies. The scope ranges from spherical shapes to worm‐like structures and up to membrane‐like vesicular objects.^[^ ^2^ ^]^ These materials have found numerous applications in the areas of drug delivery, nanoreactors, or solution templating.^[^ ^3^ ^]^ From a thermodynamical point of view, the structure evolution of the self‐assembly of block copolymers is governed by the copolymer composition, the total polymer length, and the interaction parameters between the polymers and the solvents.^[^ ^4^ ^]^ Although solution structures of different shapes are accessible, when the right conditions are applied, these structures still lack the complexity of natural systems based on proteins. In recent years, many attempts aimed to increase the complexity by the addition of noncovalent forces to the system, including crystallization,^[^ ^5^ ^]^ π‐π stacking,^[^ ^6^ ^]^ and hydrogen bonding.^[^ ^7^, ^8^ ^]^ Among the common motifs, peptides attracted considerable attention as they intrinsically possess a high tendency to force the formation of specific 1D‐morphologies due to their ability to build secondary structures based on directed hydrogen bonds. Related to this, polymer‐peptide conjugates have been frequently exploited to create anisotropic nanostructures, e.g. fibers,^[^ ^9^ ^]^ tapes,^[^ ^10^, ^11^ ^]^ or tubes.^[^ ^12^ ^]^ Their assembling tendency is strongly connected to their natural propensity to form stable secondary structures, as *β*‐sheets or *α*‐helices, in solution. Moreover, hybrid materials of polymers and peptides can benefit from the combination of the properties of both individual components.^[^ ^13^, ^14^, ^15^ ^]^ However, the integration of peptide segments in polymer structures is currently mostly limited to the conjugation of hydrophilic homopolymers, e.g. poly(ethylene oxide) (PEO),^[^ ^16^, ^17^, ^18^, ^19^ ^]^ 2‐hydroxypropyl methacrylate,^[^ ^20^, ^21^, ^22^ ^]^ whereas the attachment of peptides to block copolymers and their influence on the self‐assembly remains barely investigated.^[^ ^4^, ^12^, ^23^, ^24^ ^]^ Hence, the question remains whether the *β*‐sheet forming peptides are still able to build up secondary structures or is the aggregation altered in conjugation with a block copolymer that self‐assembles. Conversely, would the peptide interaction affect the solution morphology of the block copolymer? Non‐natural, but strong *β*‐sheet forming cyclic peptides have recently revealed an impact on the block copolymer assembly,^[^ ^24^ ^]^ but such an effect cannot be generalized for peptides and directed hydrogen bonds.^[^ ^12^, ^25^ ^]^

In this study, we want to address these questions by investigating the interaction and influence of a *β*‐sheet forming peptide sequence at the core‐shell‐interface of a self‐assembled block copolymer. For this purpose, we designed an amphiphilic diblock copolymer peptide conjugate consisting of a soft hydrophobic (PBA: poly(*n*‐butyl acrylate)) and a hydrophilic (PEO) block connected by an oligopeptide linker. The major challenge remains the localization of the comparably small peptide strains and the direct analysis of their folding state in the assembled material. To date, most characterizations of the peptide stacking relied on CD‐ and IR‐spectroscopy, providing an average evaluation of structural arrangements. Pairing with microscopy methods visualizes the resulting morphologies. However, the integration of quantitative atomic force microscopy (AFM) with AFM based tip‐enhanced Raman scattering (TERS) represents a transformative tool, capable of correlating the local topology, nanomechanical properties and chemical interactions.^[^ ^26^, ^27^ ^]^

TERS has demonstrated exceptional capabilities in localizing variations in the chemical composition and structural conformation of biomaterials at the nanometer scale with enhanced sensitivity.^[^ ^28^ ^]^ Extensive TERS studies have revealed detailed insights into polymers,^[^ ^26^, ^27^, ^29^, ^30^, ^31^ ^]^ amino acids,^[^ ^32^, ^33^ ^]^ peptides,^[^ ^34^, ^35^ ^]^ and their self‐assembled nanostructures.^[^ ^36^, ^37^, ^38^, ^39^ ^]^ High‐resolution TERS investigations of amyloid fibrils have elucidated local structural variation, advancing the understanding of their formation mechanisms and role in diseases.

By merging the molecular resolution and the chemical specificity of Raman spectroscopy,^[^ ^40^ ^]^ TERS spectroscopy provides a direct and detailed examination of the secondary structure of the oligopeptide linker within the self‐assembled PBA‐*b*‐(GFFG)‐*b*‐PEO micelles. Concurrently, advanced AFM force volume spectroscopy quantitatively assesses variations in nanomechanical properties.^[^ ^41^, ^42^ ^]^ AFM and AFM based force spectroscopy are today well‐established techniques for probing the elastic modulus and adhesion forces of peptide fibrils,^[^ ^43^, ^44^ ^]^ block copolymer micelles, and complex protein structures in tissue.^[^ ^26^, ^27^, ^45^, ^46^, ^47^, ^48^ ^]^ These approaches can yield detailed insights into their mechanical rigidity, and can provide a deeper understanding of possible assembly pathways. For instance, differences in the mechanical properties of peptide fibrils with different morphologies were attributed to changes in the internal structure.^[^ ^43^ ^]^ An increase of the elastic modulus was correlated with a higher population of *β*‐sheets.^[^ ^44^ ^]^

The combination of AFM‐TERS and AFM‐force volume spectroscopy for the investigation of the block copolymer filomicelles is anticipated to provide valuable insights into the role of the peptide shell in the self‐assembly process, and the underlying causes of observed morphological differences.

## 2 Results and Discussion

**Scheme 1:**
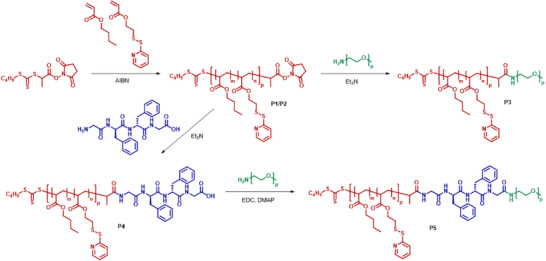
Overview of the synthetic route to create the amphiphilic block copolymers with (bottom) and without (top) the tetrapeptide linker moiety between the hydrophilic and hydrophobic block. Details on the experimental procedures and properties of the polymers P1‐5 can be found in the supporting information.

The block‐like amphiphilic conjugate was prepared by sequential coupling of the individual domains (**Scheme** **1**). The hydrophobic PBA‐block was first polymerized via reversible addition‐fragmentation chain transfer (RAFT) polymerization using a chain transfer agent (CTA), which was already functionalized with an *N*‐hydroxysuccinimide (NHS)‐ester. In order to stabilize the final nanostructure, 9 mol% of the reactive monomer pyridyl disulfide ethyl acrylate (PDSA) was copolymerized enabling the crosslinking of the final micellar core (for simplicity, the co‐monomer is not considered in the latter nomenclature). Due to the NHS‐functionality, the resulting well‐defined polymer (see SI for characteristics) could be directly coupled to the previously prepared tetrapeptide segment (GFFG) at the N‐terminus (experimental details of the solid phase peptide synthesis are given in the SI). The diphenylalanine motif is well‐known for the formation of *β*‐sheet structures, while the glycine ends were chosen to guarantee an efficient coupling to the polymers. In a subsequent step, a heterotelechelic PEO bearing an amino end group was attached to the hydrophobic peptide conjugate via activated ester coupling. After purification, the well‐defined amphiphilic conjugate PBA‐*b*‐(GFFG)‐*b*‐PEO was obtained. For comparison, an equal block copolymer lacking the peptide linker (PBA‐*b*‐PEO) was prepared by direct coupling of the amino‐PEO to the PBA. Details on the characterization of both materials can be found in the Figures S1,S2, and Table S1 (Supporting Information).

**Figure 1:**
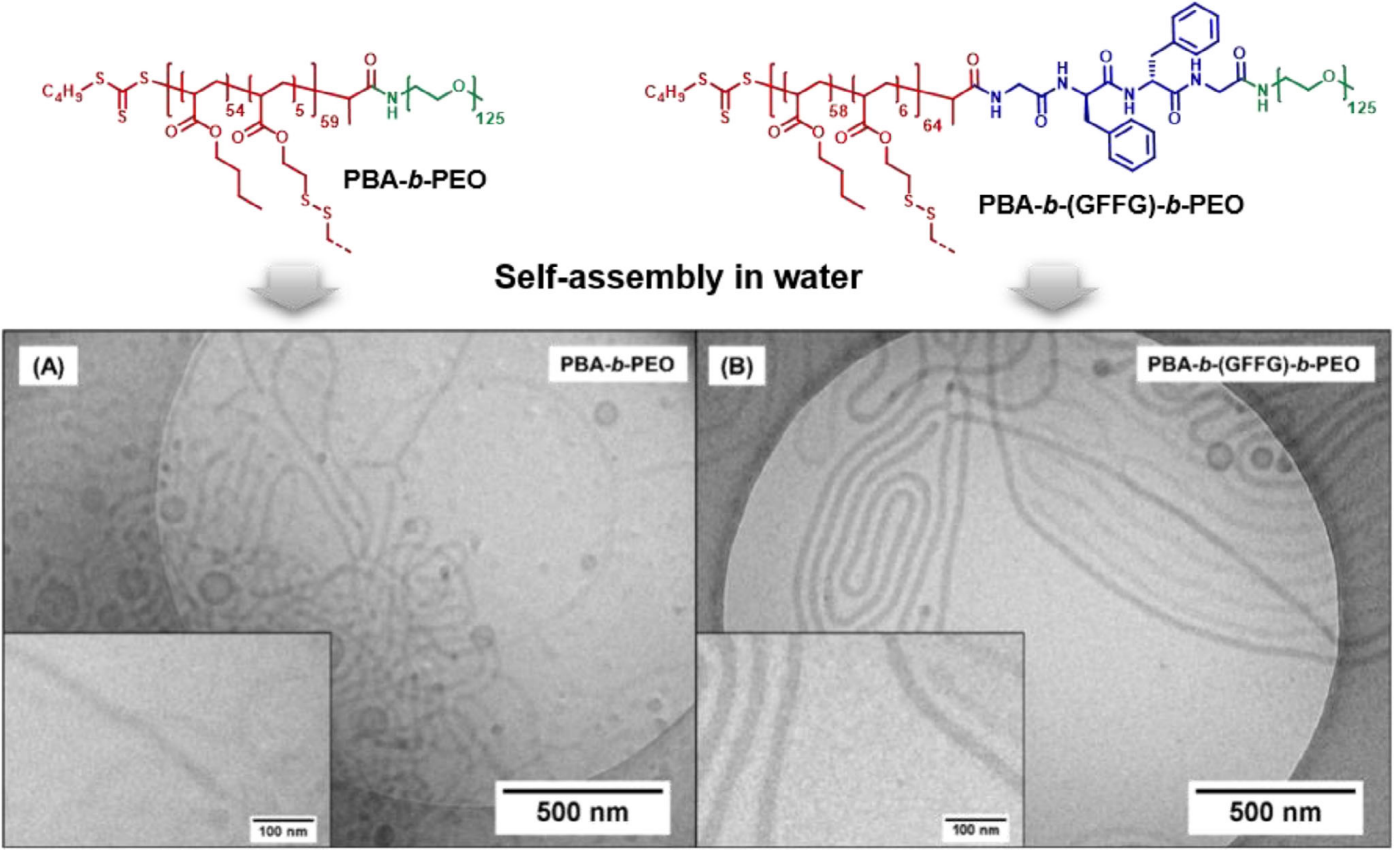
Top section: Chemical structures of the amphiphilic block copolymer (PBA‐*b*‐PEO) and the corresponding block copolymer‐peptide conjugate (PBA‐*b*‐(GFFG)‐*b*‐PEO); bottom section: cryo‐TEM images of the nanostructures formed by assembly of PBA‐*b*‐PEO A) and PBA‐*b*‐(GFFG)‐*b*‐PEO B). The inset shows a higher magnification for samples, respectively.

Subsequently, both materials (structures are depicted in **Figure** **1**A) were assembled by dissolving in DMSO as co‐solvent and a following solvent switch to water. The formed nanostructures were fixed by the addition of 1,6‐hexandithiol (HDT), which crosslinks the core by reaction with the PDSA groups. Any excess of crosslinker was removed by dialysis to obtain the final micelle dispersions in pure water. First dynamic light scattering (DLS) measurements of the resulting solutions revealed narrowly distributed structures with average hydrodynamic diameters of 107 nm for PBA‐*b*‐PEO and 157 nm for PBA‐*b*‐(GFFG)‐*b*‐PEO (Figure S3 and Table S1, Supporting Information). Despite a clear difference in size, DLS does not provide detailed information on the morphology of the nanostructures as for the calculations a spherical shape is assumed. In order to gain more detailed insights in the morphology and dimensions, the samples were further characterized by cryogenic transmission electron microscopy (cryo‐TEM) (Figure 1A,B, additional cryo‐TEM images are provided in the Figure S4, Supporting Information).

In both cases mostly wormlike structures, often called filomicelles, were obtained besides a few vesicles. The small population of vesicles might represent kinetically trapped states, due to the slow exchange dynamics in PBA‐*b*‐PEO block copolymer assemblies as previously described.^[^ ^49^ ^]^

Both filomicelles appear similar, but a closer look reveals characteristic differences in their appearance. Differences in size and core cross‐sectional diameters become apparent. Specifically, the micelles formed by peptide conjugate (diameter of 27.4 ± 3.1 nm) were found to be larger than the ones based on the plain block copolymer (diameter of 25.1 ± 2.9 nm). The determination of the contour lengths (the “end‐to‐end” length of a polymer) of the filomicelles was particular challenging for PBA‐*b*‐(GFFG)‐*b*‐PEO, as individual filomicelles exceed the limits of the cryo‐TEM images, even at low magnification. From a few fully visible structures, contour lengths of 6.5 µm and more can be estimated, while contour lengths between ≈100 nm and 2 µm are more frequently found for the block copolymer (PBA‐*b*‐PEO).

**Figure 2:**
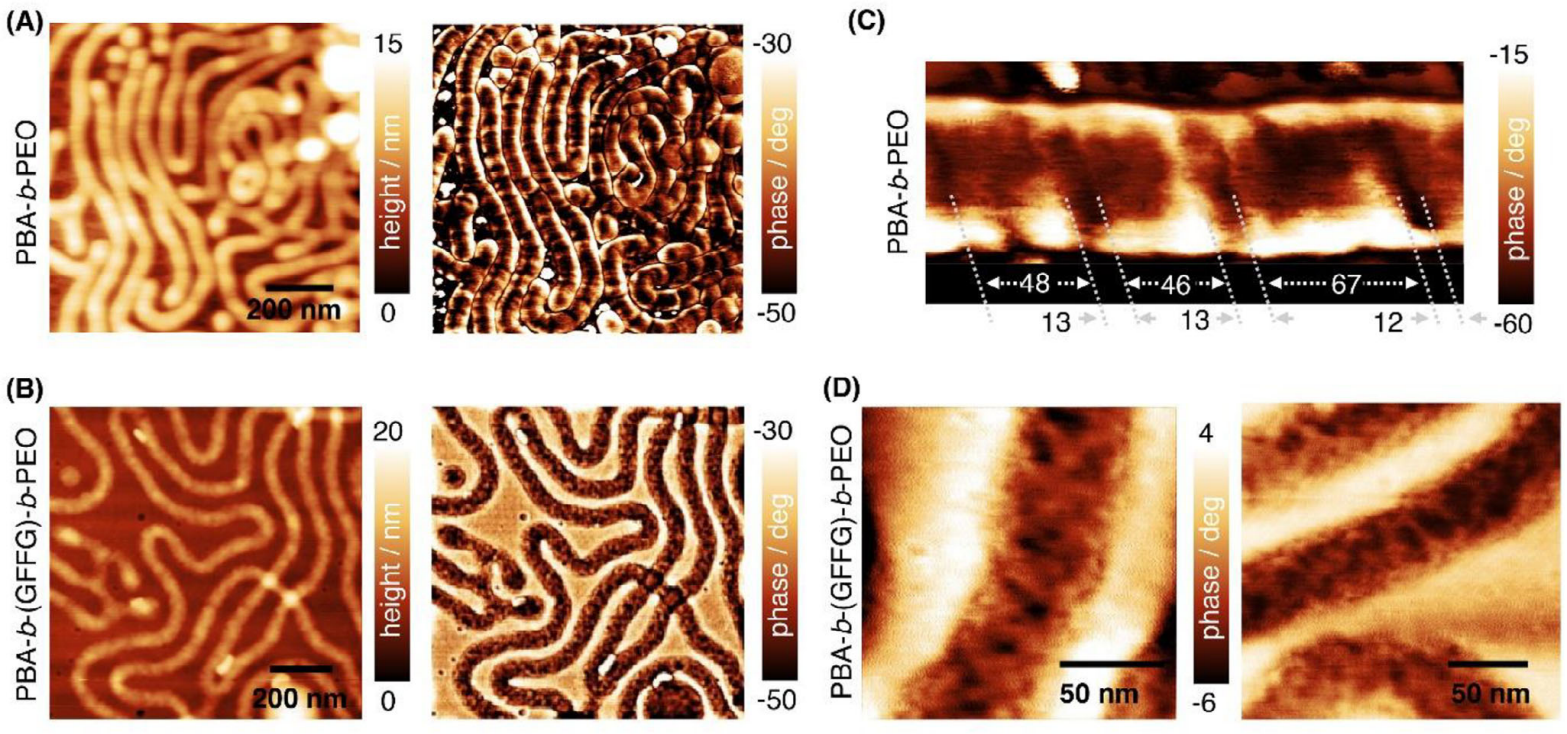
Surface morphology of filomicelles with (bottom) and without peptide shell (top). A,B) Low magnification AFM topography and phase images of the amphiphilic block copolymer (PBA‐*b*‐PEO) filomicelles (top) and of the block copolymer‐peptide conjugate (PBA‐*b*‐(GFFG)‐*b*‐PEO) filomicelles (bottom). C,D) High maginifcation AFM phase images showning the surface morphology of both types of filomicelles.

To investigate the morphological difference between the PBA‐*b*‐(GFFG)‐*b*‐PEO and the PBA‐*b*‐PEO filomicelles in greater detail, high‐resolution, large‐scale AFM topography images (**Figure** **2**; Figure S5, Supporting Information) were acquired to visualize a substantial number of individual filomicelles. The contour length of these structures was precisely measured (for details see Figure S5, Supporting Information). Consistent with the cryo‐TEM observations, the PBA‐*b*‐(GFFG)‐*b*‐PEO filomicelles exhibited a higher proportion of structures with medium to extreme contour lengths. Notably, the longest visible structures exceeded 20 µm in the contour length. On average, the contour length of these filomicelles was ≈4 (±4 µm).

In contrast, the PBA‐*b*‐PEO filomicelles did not exhibit such extreme contour lengths. The maximum contour length observed for these structures was ≈5 µm. Additionally, a significantly higher number of short (<1 µm) filomicelles is detected, resulting in an average contour length of ≈700 (±850 nm). This shift in the length distribution toward shorter contour lengths highlights substantial differences in the dimensions of the two systems, triggered only by the insertion of a tetrapeptide moiety between the hydrophobic and hydrophilic blocks of the copolymer. The significant differences in dimension of these nanostructures already implies an impact of the peptide linker on the self‐assembly process. Another, yet a more subtle difference is found in the general appearance of the respective nanostructures. While the filomicelles formed by the block copolymer seem to be flexible and frequent coiling is observed, the peptide conjugate forms rather elongated patterns with parallel alignments (see TEM images, Figure 1). The low glass transition temperature (T_g_) of the PBA and the crosslinking should render the core rubber‐like, which supports a flexible configuration as seen for PBA‐*b*‐PEO. The more rigid appearance of the PBA‐*b*‐(GFFG)‐*b*‐PEO nanostructures might therefore relate to a peptide interaction.

To further investigate the mechanical behaviour of both filomicelles, the wormlike chain model (WLC) was employed to determine the persistence length (see Suppl. Information),^[^ ^50^ ^]^ providing insights into their mechanical rigidity (Figure S5b and Table S2, Supporting Information). Using a representative dataset of over 70 filomicelles for each sample, the persistence lengths were determined to be 132 nm (±53) nm for the PBA‐*b*‐PEO micelles and 152 nm (±53) for the PBA‐*b*‐(GFFG)‐*b*‐PEO filomicelles. In both cases, the persistence lengths are significantly smaller than the contour lengths, indicating that both types of filomicelles possess a high flexibility. Notably, the PBA‐*b*‐(GFFG)‐*b*‐PEO filomicelles display a slightly larger persistence length compared to the control sample PBA‐*b*‐PEO lacking the tetrapeptide moiety. These results suggest a modest increase in the mechanical robustness despite the overall high flexibility.

The PBA‐*b*‐(GFFG)‐*b*‐PEO filomicelles exhibit slightly higher bending rigidity compared to the PBA‐*b*‐PEO filomicelles, resulting in reduced flexibility. This aligns with the greater bending capacity of PBA‐*b*‐PEO filomicelles observed in TEM and AFM images, where small ring‐like structures are frequently present. Despite their higher flexibility, the axial elastic modulus of PBA‐*b*‐PEO filomicelles is significantly higher, indicating that they are intrinsically stiffer than the PBA‐*b*‐(GFFG)‐*b*‐PEO filomicelles. These differences suggest that the intrinsic stiffness is primarily determined by the properties of the PBA‐PDSA core, particularly its crosslinking efficiency, whereas the bending rigidity is more strongly affected by the mechanical properties of the peptide shell.

The calculated elastic moduli of 0.9 (±0.6 MPa) for PBA‐*b*‐(GFFG)‐*b*‐PEO and 2.7 (±1.4 MPa) for the for PBA‐*b*‐PEO filomicelles are higher compared to the Young's modulus of PBA (≈0.1 MPa), implying a moderate crosslinking rate.^[^ ^51^ ^]^ Notably, the thin peptide shell does not seem to significantly contribute to the elasticity. Instead, the peptide shell may slightly affect the crosslinking efficiency in the PDA‐PDSA core, potentially due to an altered interface that limits the diffusion of the added crosslinker into the core of the micelles.^[^ ^52^ ^]^ As a result, the reduced crosslinking density leads to a modest reduction in the elastic modulus of the PBA‐PDSA core in PBA‐*b*‐(GFFG)‐*b*‐PEO filomicelles. Conversely, the peptide shell slightly increases the bending rigidity of PBA‐*b*‐(GFFG)‐*b*‐PEO filomicelles, probably due to the formation of ordered secondary structures along the circumference of the core, e.g., the presence of *β*‐sheet structures that likely impart rigidity.

At first glance, these mechanical properties seem to contradict the observation of extremely long PBA‐*b*‐(GFFG)‐*b*‐PEO filomicelles. Typically, a lower elastic modulus and higher bending rigidity would reduce the mechanical stability of the structures, decreasing their ability to accommodate local packing constraints or adopt curved configurations. However, the interactions of the peptide moieties at the interface might enhance thermodynamic as well as kinetic factors that impede the formation of stable configurations. Maintaining the structural integrity of these filomicelles might require subtle adjustments in bending rigidity to minimize defects and stabilize the highly dynamic behavior of the block copolymers, thereby supporting the formation of long, ordered structures. Additionally, the interfacial energy / interfacial tension at the core‐corona interface plays a critical role in micelle morphology. A lower interfacial energy typically promotes the formation of highly curved structures. By introducing a peptide layer between the hydrophobic PBA‐PDSA core and the hydrophilic PEO corona, the interfacial energy should be reduced, and thus, facilitate the formation of worm‐like micelles.

The microscopic morphology of the PBA‐*b*‐PEO and PBA‐*b*‐(GFFG)‐*b*‐PEO filomicelles observed in high resolution AFM investigations resembles the one found in cryo‐TEM studies. Despite the strong agreement between cryo‐TEM and AFM observations at the microscopic scale, high‐magnification AFM phase images (Figure 2) reveal distinct nanoscale surface morphologies that are not discernible in the cryo‐TEM investigations. Specifically, PBA‐*b*‐PEO filomicelles frequently exhibit a smooth, block‐like pattern with segment lengths of ≈50–70 nm, separated by pitches of 10–15 nm. In contrast, PBA‐*b*‐(GFFG)‐*b*‐PEO filomicelles display a predominantly regular, filamentous surface structure, although faint block‐like patterns are occasionally discernible beneath this filamentous morphology.

The incorporation of four amino acids in the interfacial shell region of PBA‐*b*‐(GFFG)‐*b*‐PEO results in only minor height variations (≈1–2 nm). On average, both samples exhibit similar heights, with PBA‐*b*‐PEO measuring ≈5 nm and PBA‐*b*‐(GFFG)‐*b*‐PEO measuring ≈7 nm. Typically, these values are much smaller than those observed in cryo‐TEM images, due to the drying steps required for AFM sample preparation, leading to a collapse of the otherwise hydrated PEO shell, and due to PEO shell substrate interactions, which also result in a slight compression of the PEO shell.

The differences in the surface morphology and the contour length between the PBA‐*b*‐(GFFG)‐*b*‐PEO and the PBA‐*b*‐PEO filomicelles are potentially imposed by the intermediate peptide shell of the PBA‐*b*‐(GFFG)‐*b*‐PEO filomicelles, which may organize into ordered secondary structures, and may form noncovalent hydrogen bonding and π–π‐stacking interactions via the phenylalanine residues. In‐depth information on the nanoscale surface morphology of the assembly can be obtained from TERS investigations, which enable distinguishing ordered and disordered peptide regions with nanoscale resolution and high sensitivity. As a surface sensitive method, TERS primarily provides information on the PEO corona and the shell‐core interface. Consequently, TERS is well suited to differentiate the PBA‐*b*‐(GFFG)‐*b*‐PEO from PBA‐*b*‐PEO filomicelles by identifying specific marker bands associated with the crosslinked core and the intermediate peptide interface.

**Figure 3:**
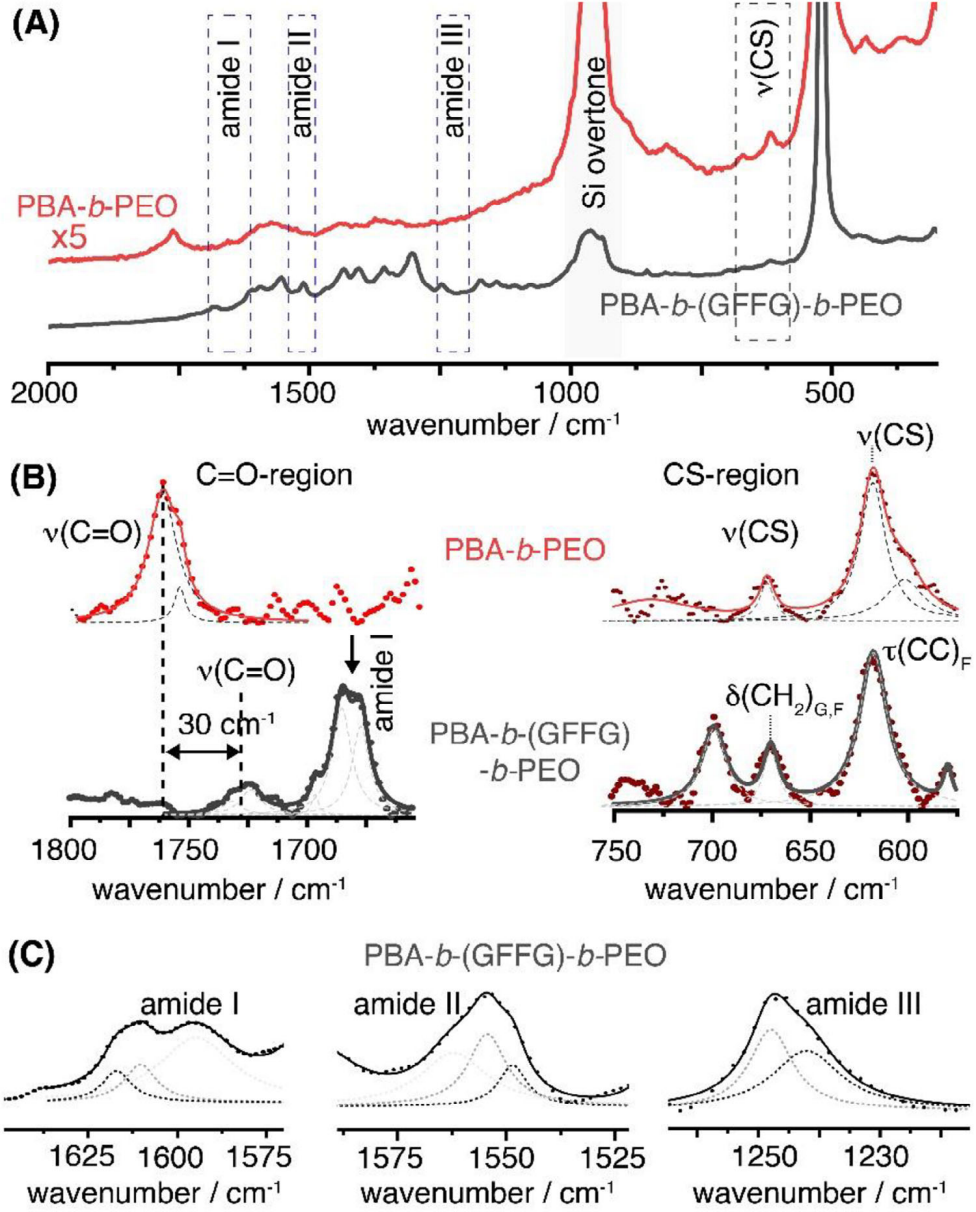
a) Average TERS spectrum recorded across a larger area of filomicelles at a laser irradiation power P_inc_≈ 200‐500 µW. b) Comparison of the TERS peak pattern in the carbonyl region (left) and the CS‐region (right) for PBA‐*b*‐PEO (red) and PBA‐*b*‐(GFFG)‐*b*‐PEO (grey) filomicelles. Red and black dots correspond to the raw data, solid red and black lines show the resulting cumulative fits according to the applied band decomposition as indicated by the grey dotted lines. c) Shape and band position of the amide I‐III band associated with the secondary structure formation of the peptide shell present in the average spectrum of the PBA‐b‐(GFFG)‐b‐PEO filomicelles. Black dots represent raw data points, solid black line corresponds to the cumulative fit based on the peak decomposition (grey doted lines).

**Figure** **3**a presents the average TERS spectra recorded for both filomicelles. Raman band assignments for the most prominent functional groups and structural motifs are provided in Table S3 (Supporting Information). The TERS spectrum of the PBA‐*b*‐PEO filomicelles (Figure 3a, red spectrum) reveals only a few weak Raman bands, primarily corresponding to the ν(C‐S) and ν(C═O) stretching modes, which can be attributed to the PBA/PDSA core. In contrast, the average TERS spectrum of PBA‐*b*‐(GFFG)‐*b*‐PEO filomicelles exhibits a richer pattern of Raman bands. Here, Raman bands occur in PBA/PDSA silent regions, and can be assigned to the amide I, II and III peptide bands, as well as characteristic Raman bands of the amino acid side chains, particularly the F_i_‐modes of the phenylalanine.^[^ ^53^ ^]^ However, averaging across a larger number of filomicelles results in significant broadening of these bands, with full‐width at half maximum (FWHM) values exceeding 25 cm^−1^ (Figure 3c). This broadening is particularly evident for the amide III band at ≈1250 cm^−1^, the amide II band at ≈1550 cm^−1^ and the amide I band found in the high wavenumber region (>1660 cm^−1^). The asymmetry of these bands may arise from averaging over different secondary structures within the assemblies. Additionally, overlapping Raman bands associated with the phenylalanine residues may contribute to the amide band broadening.

Changes are also evident for the core‐specific bands. Particularly, Raman bands associated with the crosslinked core (ν(C‐S)) cannot be uniquely assigned in the PBA‐*b*‐(GFFG)‐*b*‐PEO filomicelles (Figure 3b). Raman peaks in the 600–700 cm^−1^ region are more likely assigned to amino acid specific bands. Based on the position and symmetry, these Raman bands correspond to the CC torsion mode of phenylalanine and the C‐H_2_ deformation band of glycine. Raman bands associated with the PEO shell can be assigned to the (γ(CH_2)_ +ν(CO) combination bands at ≈820 cm^−1^/≈880 cm^−1^ and the ν(CO) band at ≈1080 cm^−1^/≈1124 cm^−1^, accordingly to a trans or gauche CO bond conformation. However, these bands are usually very weak, and thus, particularly the combination bands in the 800–900 cm^−1^ spectral regions can be obscured by the strong Si overtone band (900‐1000 cm^−1^). Additionally, a significant variation (Δν ≈30 cm^−1^) in the vibrational frequency of the ν(C═O) stretching mode is observed (Figure 3b). This variation suggests probing of different chemical groups, environments, or interactions, as the C═O band position can be strongly affected by electronic effects, hydrogen bonding, matrix effects, conformational and steric influences.^[^ ^54^ ^]^

For the PBA‐*b*‐PEO filomicelles, the C═O band exhibits its characteristic shape due to overlapping symmetric and antisymmetric stretching modes, and appears at higher wavenumbers (ν(C═O) ≈1756/1760 cm^−1^). This indicates that the signal predominantly stems from the strengthened C═O bond in the O═C─NH bonds present in the transition region between the PBA‐PDSA block and the PEO block. In contrast, the PBA‐*b*‐(GFFG)‐*b*‐PEO filomicelles display only a faint signature of the polymer‐associated C═O bands at lower wavenumbers (ν(C═O) ≈ 1724/1732 cm^−1^), implying a reduced bond strength. This signal likely originates from the PBA core region, which is also supported by the lower relative intensity of the band indicating an enlarged distance to the TERS tip. The low intensity suggests that these functional groups are buried underneath a peptide shell. Furthermore, the appearance of a pronounced band at ≈1680 cm^−1^ is consistent with the amide I band of GFFG‐peptide moieties. The band position is characteristic for *β*‐strands and *β*‐sheets, further supporting the hypothesis that the peptide shell governs the observed spectroscopic features.

The average TERS spectra demonstrate the exceptional sensitivity of TERS in identifying nanoscale chemical and structural differences, as the corresponding TERS signal originates only from presumably a peptide monolayer formed by the tetrapeptide linker GFFG wrapping around the elongated micellar core. Despite the short peptide length, the average TERS spectrum cannot be unequivocally assigned to a specific secondary structure. Instead, the broadened amide Raman bands suggest the presence of a mixture of secondary structures, which can arise from intermolecular hydrogen bonding even for oligo‐peptides. Based on the observed peak widths and asymmetry (see Figure 3c), the amide bands seem to be composed of multiple contributions. Spectral decomposition implies that both unordered and ordered structures might be present, according to the observed amide I and amide III band shifts. However, the average TERS spectrum does not provide detailed insights into the specific types of secondary structures contributing to the signal or their spatial distribution along the filomicelles. To gain this level of detail, spatially resolved TERS investigations of individual filomicelles are required.

**Figure 4:**
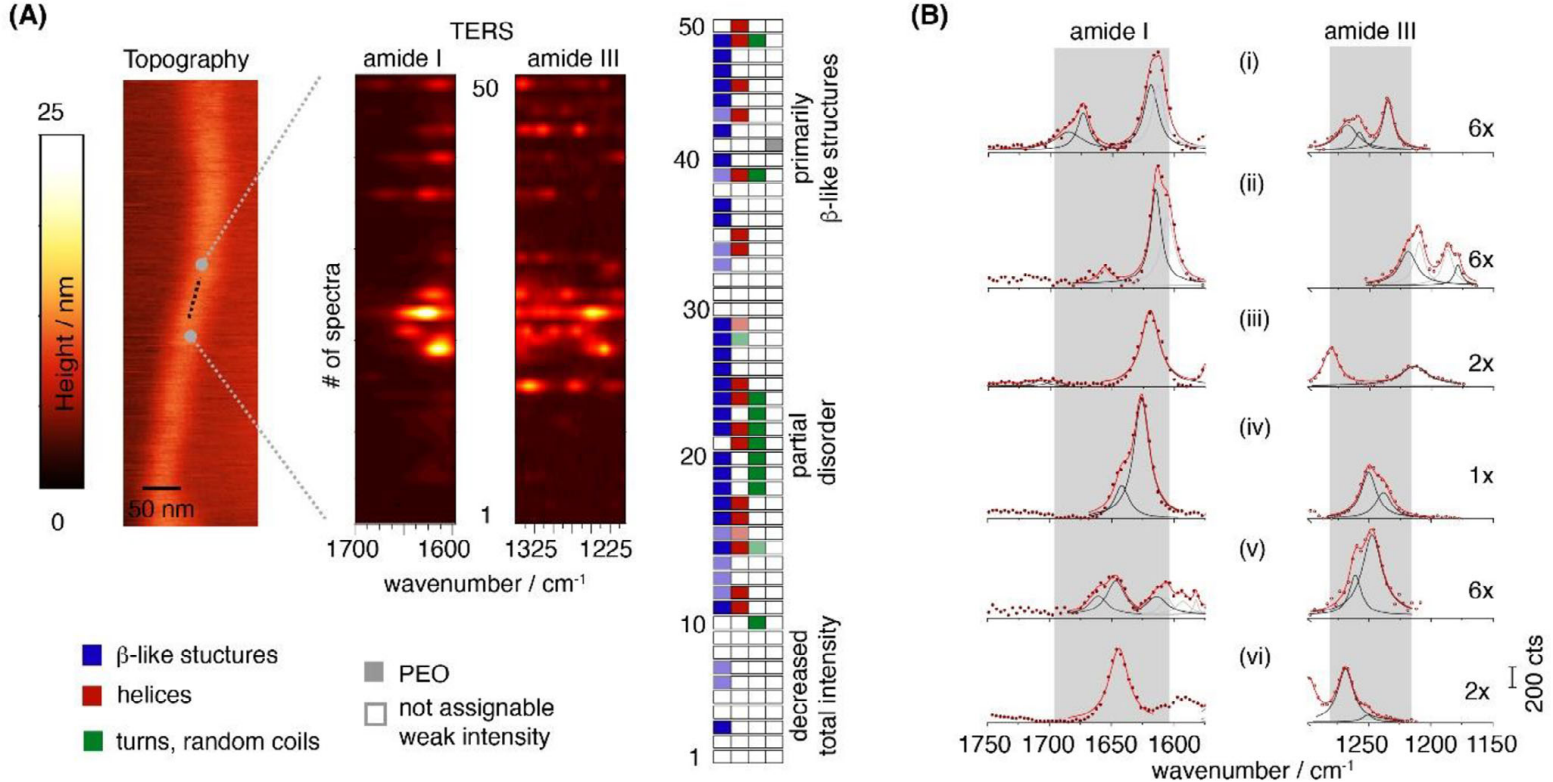
TERS investigation of individual (PBA‐*b*‐(GFFG)‐*b*‐PEO) filomicelles. a) TERS lineprofile comprising 50 TERS spectra recorded at a distance of 1 nm in the center of the filomicelles (dashed line in the topography image). TERS spectra are shown for the amide I and amide III region as waterfall plots (middle). Assignment of the secondary structure according to the observed Raman peak pattern, i.e., Raman band position, (left). Light blue and red square correspond to weak signatures. TERS spectra covering the entire spectra fingerprint region are provided in Figure S8 (Supporting Information). b) Amide I and amide III region of individual spectra exemplifying the presence of different secondary structures (diamonds, solid and open circles correspond to the background subtracted data, red solid lines represent the cumulative fit resulting from Raman band decomposition by multiple peak fitting (grey dashed lines). Spectra (i) to (vi) correspond to positions 2, 10, 25, 27, 28, 29 of the lineprofile shown in (A). TERS spectra acquisition time: 1s; incident laser power at the sample P_inc_≈ 500 µW.

To investigate the occurrence of different structural conformations in more detail, TERS mapping was performed along the contour length of individual filomicelles. TERS spectra were recorded at intervals of 1 nm in the center of a filomicelle. **Figure** **4**a illustrates the TERS investigation of a selected region of interest, specifically adjusted to a straight, homogenous segment of an extended (PBA‐*b*‐(GFFG)‐*b*‐PEO) filomicelle. The assignment of different peptide conformations was based primarily on the band positions in the amide I and amide III regions, with additional consideration of the amide II band. Figure 4a shows waterfall plots of the amide I and amide III regions along the selected line outlined in the topography image. The mapping reveals a complex peak pattern that varies along the line profile, including shifts in the band position and the overall TERS spectral intensity. A detailed analysis of these variations is exemplified in Figure 4b, which displays close‐ups of the amide I and amide III regions along with band deconvolutions, enabling identification of secondary structures. The top spectrum (Figure 4b‐(i)) clearly indicates the presence of *β*‐sheets, as evidenced by the characteristic band positions (ν_AmIII_ = 1234 cm^−1^, ν_AmI_ = 1619 cm^−1^ and 1673 cm^−1^, ν_AmII_ = 1529 cm^−1^). A broad band at higher wavenumbers in the amide I region (ν_AmI_ = 1673/1686 cm^−1^) suggests the coexistence of *β*‐strands and helices. The presence of helices is further supported by a Raman peak in the amide III region, potentially corresponding to 3,_10_‐helices or partially distorted, non‐standard helices (ν_AmIII_ = 1268 cm^−1^)). Similarly, the TERS spectrum shown in Figure 4b‐(ii) also indicates the presence of *β*‐sheets. Apparently, the amide I and amide III bands are shifted to lower wavenumbers (ν_AmIII_ = 1219 cm^−1^, ν_AmI_ = 1614 cm^−1^ and 1669 cm^−1^). Additionally, the TERS spectrum also reveals typical bands associated with the phenylalanine residue, i.e., the phenyl ring bond CC stretching (F_1_ mode at 1605 cm^−1^) and its coupling to the CC stretching vibration (F_3_ mode at 1210 cm^−1^).

**Figure 5:**
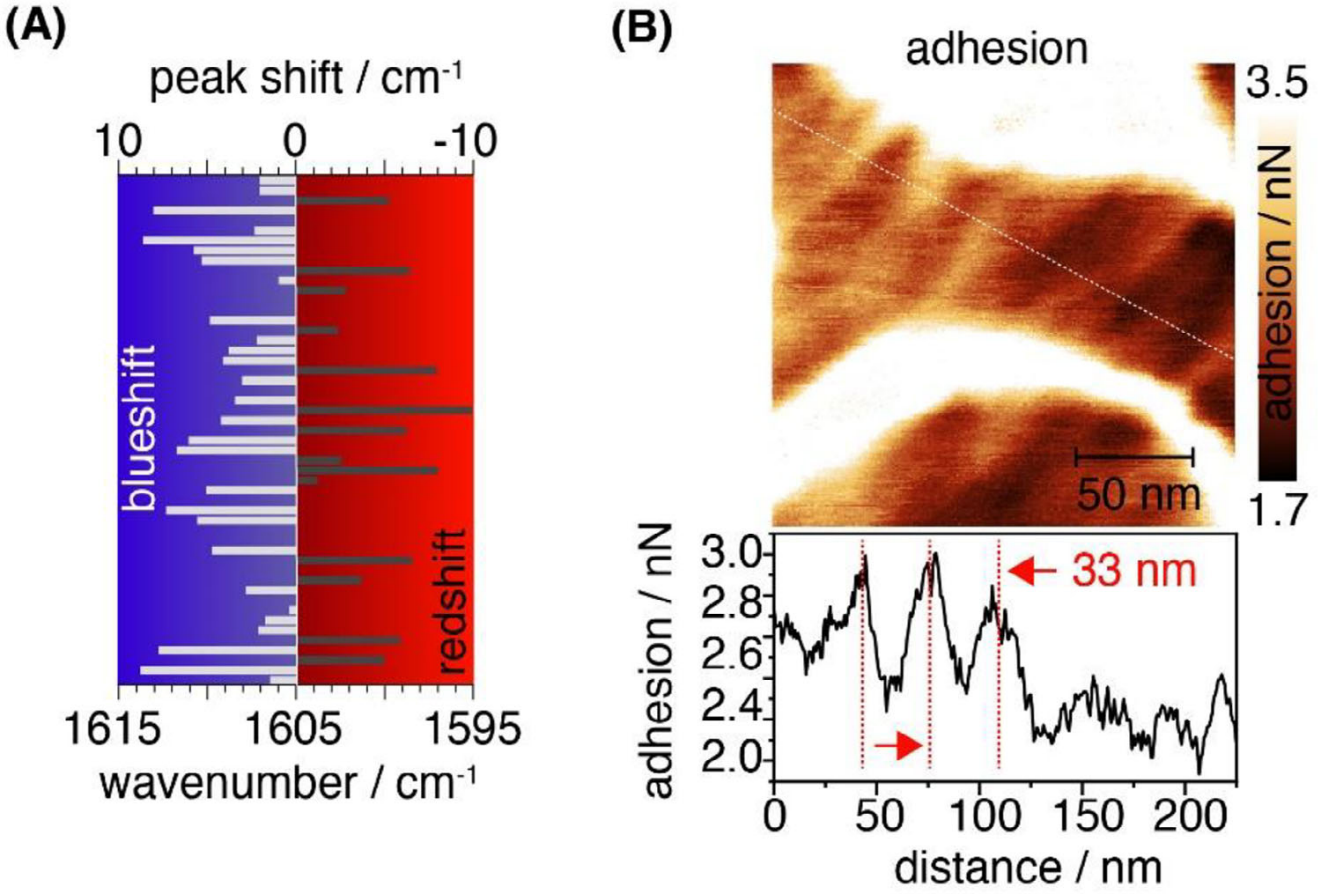
a) Bandshift of the F5 mode observed in individual TERS spectra of PBA‐*b*‐(GFFG)‐*b*‐PEO filomicelles reveals blue‐ and redshifts relative to the expected band position at 1605 cm^−1^. b) High magnification AFM adhesion map (top) recorded on an individual PBA‐*b*‐(GFFG)‐*b*‐PEO filomicelle and cross‐section of the adhesion force (bottom) along the dotted white line showing small alternating variations in the adhesion force along the contour length of the filomicelles.

The observed shifts of the amide bands to the lower wavenumber regions are characteristic for strong hydrogen bonding, resulting in the formation of highly ordered *β*‐sheets. The band position at 1619 cm^−1^ further implies either a high content of antiparallel *β*‐sheets or the presence of strained beta sheets. Such strained structures can, e.g., arise from twists, kinks or mechanical stress due to the filomicelle core's curved geometry, forcing the *β*‐sheets to adopt the radius of curvature. A suppression of the amide II band, which may result from specific alignment or tight packing, would be consistent with a high rigidity. Figure 4b‐(iii) displays a spectrum, with bands at 1213, 1281, and 1619 cm^−1^, which indicates a strong contribution of *β*‐sheets, which is also supported by the amide II band at 1520 cm^−1^. The strong amide III band at 1281 cm^−1^ might be indicative for the occurrence of non‐standard helices. Figure 4b‐(iv) presents a TERS spectrum where the amide I and amide III bands exhibit significant asymmetry. Decomposition of these bands reveals two distinct contributions: one associated with (parallel) *β*‐sheets, (ν_AmIII_ = 1238 cm^−1^, ν_AmI_ = 1626 cm^−1^) and the other random coils (ν_AmIII_ = 1250 cm^−1^, ν_AmI_ = 1642 cm^−1^). **Figure** **5**b‐(v,vi) displays TERS spectra characteristic for a high prevalence of helical secondary structures, as the amide III and amide I bands shift to even higher wavenumbers (ν_AmI_ ≈1644–1660 cm^−1^ and ν_AmIII_ ≈1260–1280 cm^−1^). These shifts are indicative for α‐helices and non‐standard helices, the latter being frequently observed in short peptides.

Based on the observed amide peak pattern, each spectrum was assigned to the most likely secondary structures. Apparently, most spectra can be assigned to a mixture of secondary structures. In the presented example, the recorded TERS spectra predominately exhibit *β*‐like structures, i.e., *β*‐sheets and *β*‐strands (≈80% of the displayed spectra of Figure 4a), as well as helical structures (≈34% of all spectra of Figure 4a). The assignment of the amide bands highlights the presence of highly ordered nanoscale regions, predominantly composed of *β*‐sheets, *β*‐strands and helices, interspersed with partially disordered regions, as deduced by the presence of random coils, and potentially nonstandard helices. Within the analyzed area, the ordered regions span several nanometers (≈10–15 nm). Rather than being completely disordered, the backbone of short oligopeptides can adopt certain secondary structures. For example, molecular dynamics (MD) simulations have demonstrated that the tripeptide GFG can adopt *β*‐strands, polyproline‐II (pP‐II) helices, helical, classical, and inverse γ‐turns with varying populations.^[^ ^55^ ^]^ The distribution of these conformations depends on the chemical nature of the involved residues. For glycine‐rich tripeptides major contributing secondary structures are *β*‐like and pP‐II helical conformations. Therefore, the observed coexistence of *β*‐like and helical secondary structures for the PBA‐*b*‐(GFFG)‐*b*‐PEO filomicelles is reasonable. Throughout a series of TERS investigations (# > 5) utilizing either line profiling or TERS mapping (see Figure S9, Supporting Information), we find a high reproducibility in terms of the coexistence of *β*‐sheet, helical and partially disordered structures, which is also in good agreement with the uniformity of the spectra shown in Figures S6 and S7 (Supporting Information) and additional data displayed in Figure S9 (Supporting Information). Furthermore, no apparent changes were observed with respect to their morphology and secondary structure formation in the peptide shell of the PBA‐*b*‐(GFFG)‐*b*‐PEO filomicelles, which showed a high long‐term stability (>3 years).

Peptide aggregation into highly structured morphologies, e.g., fibrils, is hypothetically facilitated by stabilizing π‐π‐stacking of aromatic rings of the phenylalanine residues promoting directional assembly.^[^ ^56^ ^]^ The role of π‐π‐stacking in self‐assembly processes has been discussed in terms of increased growth rates and enhanced thermodynamical stability of the resulting structures. Similarly, the self‐assembly process of the block‐copolymer PBA‐*b*‐(GFFG)‐*b*‐PEO into extended filomicelles may benefit from π‐π‐stacking in the peptide shell.

The vibrational frequencies of the F_i_ modes associated with phenylalanine residues are sensitive to molecular interactions,^[^ ^55^ ^]^ making them valuable indicators of local environmental and structural organization. π‐π stacking interactions, e.g., cause electron redistribution and alter vibrational coupling, leading to a reduction in bond strength and a corresponding redshift in the vibrational frequencies of the ring‐associated F_i_ modes. The extent of this redshift correlates with the interaction strength. To evaluate potential π‐π stacking, the F_1_ band, typically observed at 1605 cm⁻^1^, was carefully analyzed across several individual TERS spectra.

Figure 5a shows the F_1_ Raman band position and associated shift. In a few instances, a redshift of the F_1_ mode (Δν_F1_ ≤ 10 cm^−1^) was observed, consistent, with the typically observed magnitudes of π‐π stacking‐induced shifts (≈5–10 cm^−1^).^[^ ^57^ ^]^ While these shifts indicate the presence of π‐π stacking interactions, they appear to be spatially localized. More frequently, a blueshift of the F_1_ mode was observed, with shifts as large as 10 cm^−1^. These blueshifts seem to contradict a major role of π‐π‐stacking interactions in the self‐assembly process.

Instead, these blueshifts may result from other environmental factors, such as interactions with non‐polar entities that slightly increase the electron density in the aromatic ring or from increased rigidity imposed by strong hydrogen bonding in the surrounding structure, i.e., near the phenylalanine residues.

As the peptide interfaces the hydrophobic core, the phenylalanine residues might interact with the non‐polar moieties, particularly if the aromatic ring rotates toward the core region. High magnification AFM phase images of the PBA‐*b*‐(GFFG)‐*b*‐PEO filomicelle (Figure 2) revealed a highly structured surface morphology. While phase changes comprise information of the nanomechanical properties, they usually cannot be separated and quantified from the phase shift itself. To investigate the potential impact of localized polarity changes in the environment of the phenylalanine residues adhesion forces were probed through AFM tip interactions in force‐volume measurements. Quantitative adhesion maps of the PBA‐*b*‐(GFFG)‐*b*‐PEO filomicelles (Figure 5b) clearly show localized, alternating adhesion forces, which correlate with the surface morphology observed in the AFM phase images. This suggests that the observed blueshift of the F_1_ mode may be, at least in part, caused by these local polarity fluctuations. However, the significant shift of up to 10 cm^−1^ implies that potentially also other factors contribute to this effect.

The TERS investigations reveal that the peptide interface of the PBA‐*b*‐(GFFG)‐*b*‐PEO filomicelles is predominantly assembled into highly ordered secondary structures, with *β*‐like structures representing the major contribution. The robust hydrogen bonding in these structures might create a more rigid environment potentially constraining vibrations in the phenylalanine side chains or facilitating vibrational coupling between adjacent phenylalanine residues, the backbone or moieties associated with the block copolymer core and corona. The extent of the band shifting depends on the relative orientation of the hydrophobic moieties and their packing density and stacking geometry. Consequently, rigidity‐induced changes are often marked by blueshifts in the F_i_ modes. Instead, vibrational coupling may induce either redshifts or blueshifts.

It is likely that the hydrophobic phenylalanine moieties exhibit a higher affinity toward the hydrophobic core, with the phenylalanine rotating toward the core, while the peptide backbone shields them from the hydrophilic PEO corona. Interestingly, in most recorded TERS spectra the F_5_ mode – associated with the ring breathing mode of the phenylalanine side chain – is absent, despite the presence of other F‐modes. This absence suggests that the aromatic rings of the phenylalanine residues adopt an in‐plane orientation, i.e., parallel to the circumference of the peptide interface and has been observed previously.^[^ ^33^ ^]^ In such an orientation, the rings are not efficiently excited by the longitudinal electromagnetic field generated at the tip apex. Moreover, correlating the secondary structure information with the observed F_1_ band shift reveals notable trends. Spectra implying a high content of *β*‐sheets often exhibit at the same time a large blueshift of the F_1_ band. In contrast TERS spectra assigned to largely helical structures usually show minor blueshifts, or significant redshifts.

## 3 Conclusion

In conclusion, modifying the properties of the core‐shell interface by inserting a tetrapeptide linker between the PBA‐core and the PEO corona, results in filomicelles with significant morphological differences and noticeable changes of their properties. While both block copolymers, *i.e*., with and without peptide linker, self‐assemble into elongated worm‐like micelles at the given composition, the peptide linker introduces interfacial properties promoting the formation and stabilization of filomicelles with extremely long contour lengths. The elasticity of the micelles appears not to be affected majorly by the tetrapeptide moiety, but persistence length of the filomicelle with the peptide linker is increased compared to the ones derived from the simple block copolymer. TERS investigations evidence that the interfacial peptide shell is predominantly composed of *β*‐sheets and helices. This indicates that noncovalent hydrogen bonding acts as the major additional driving force in the assembly process, resulting in a sixfold extension of their contour length on average, similar to previous observations for nanofibers formed of peptide amphiphiles.^[^ ^58^ ^]^ The introduction of the tetrapeptide moiety in between both blocks therefore creates an at least partially ordered peptide shell around the core, which appears to be associated with an increase in the bending rigidity due to the formed *β*‐sheets but at the same time a decrease of the elastic modulus of the core itself. The lower elastic modulus likely arises from a lower degree of crosslinking which can be related to hindered diffusion of the added crosslinker through the peptide shell. Nevertheless, both higher bending rigidity and lower stiffness can decrease the interfacial tension, resulting in a longer contour length. Our results show that, despite the presence of strong interfacial forces, the self‐assembly of block copolymers can be modified by introducing additional secondary interaction forces at the interface of both blocks, opening up interesting avenues for tuning the properties of the nanostructures. The combination of atomic force microscopy and TERS appears to be a powerful tool for revealing these properties and providing information on interfacial structures.

## Conflict of Interest

The authors declare no conflict of interest.

## Author Contributions

The manuscript was written through the contributions of all authors. All authors have given approval to the final version of the manuscript.

## Supporting information



Supporting Information

## Data Availability

The data that support the findings of this study are available from the corresponding author upon reasonable request.
