# Supplementary material for: Unraveling the Hierarchical Self‐Assembly of Amphiphilic Block Copolymer‐Peptide Conjugates by Tip‐Enhanced Raman Spectroscopy
Source: Small. 2025 Jun 16;21(32):2502157. doi: 10.1002/smll.202502157 (PMC12366249; doi:10.1002/smll.202502157)
Supplement: Supplementary file 1 — Supporting Information [file SMLL-21-2502157-s001.docx]

**Supporting information**

Unraveling the Hierarchical Self-assembly of Amphiphilic Block Copolymer-peptide Conjugates by Tip‐Enhanced Raman Spectroscopy

Christiane Höppener,^a,b,c*^ Fabian H. Sobotta,^c,d,#^  Stephanie Hoeppener,^c,d^

Volker Deckert,^a,b,c^ Johannes C. Brendel^e,f*^

^a^ Leibniz Institute of Photonic Technology (IPHT) Albert-Einstein-Straße 9, D-07745 Jena, Germany

^b^ Institute of Physical Chemistry (IPC) and Abbe Center of Photonics, Friedrich Schiller University Jena, Helmholtzweg 4, 07743 Jena, Germany

^c^Jena Center for Soft Matter (JCSM), Friedrich Schiller University Jena, Philosophenweg 7, 07743 Jena, Germany

^d^Laboratory of Organic and Macromolecular Chemistry (IOMC), Friedrich Schiller University Jena, Humboldtstrasse 10, 07743 Jena, Germany

^e^Macromolecular Chemistry I, University of Bayreuth, Universitätsstr. 30, 95447 Bayreuth, Germany

^f^Institute of Macromolecular Research (BIMF) and Bavarian Polymer Institute (BPI), University of Bayreuth, Universitätsstr. 30, 95447 Bayreuth, Germany

*Correspondence to C. Höppener (Christiane.Hoeppener@leibniz-ipht.de), or J. C. Brendel (johannes.brendel@uni-bayreuth.de)

^#^ Current address: Institute for Complex Molecular Systems, Department of Chemical Engineering and Chemistry, Eindhoven University of Technology, P.O. Box 513, 5600 MB Eindhoven, Netherlands

**Experimental part**

***Materials and Methods***

All chemicals and solvents were purchased from Sigma-Aldrich, Iris Biotech, Acros Organics and if not otherwise mentioned, were used without further purification. Pyridyldisulfide ethyl acrylate (PDSA) and 2-(butylthiocarbonothioylthio)propanoic hydroxysuccinimide (PABTC-NHS) were prepared *via* previously reported procedures.[[1](#_ENREF_1), [2](#_ENREF_2)] 1,4-dioxane and butyl acrylate (BA) were treated 24 h with inhibitor remover resin prior to use. mPEO-NH_2_ was purchased by Rapp Polymere. ^1^H-NMR was performed at room temperature on a Bruker AC 300 MHz spectrometer. Size-exclusion chromatography (SEC) of the polymers was performed on a Shimadzu system equipped with an SCL-10A system controller, an LC-10AD pump, a RID-10A refractive index detector and a PSS SDV column with *N*,*N*-dimethylacetamide (DMAc) + 0.21% LiCl as eluent. The column oven was set to 50 °C. Dynamic light scattering (DLS) was performed on a ZetaSizer Nano ZS (Malvern, Herrenberg, Germany) equipped with a He–Ne laser operating at a wavelength of λ = 633 nm. Counts were detected at an angle of 173°. The particle size was approximated as the effective diameter (Z-average) obtained by the cumulants method assuming a spherical shape. All measurements were conducted at 25 °C in semi-micro cuvettes after equilibration times of 30 s in triplicate. Every measurement included 10 runs, in which every run took 30 seconds. Apparent hydrodynamic radii were calculated using the Stokes-Einstein Equation (1):

$R\text{h}=\frac{kT}{6\pi\eta D}$ (1)

R_h_ = hydrodynamic radius, k = Boltzmann constant, T = absolute temperature, η = viscosity of the sample and D = apparent translational diffusion coefficient.

***Procedure for the solid phase peptide synthesis***

The synthesis was proceeded according to literature known procedure.[[2](#_ENREF_2)] 2-chlorotrityl resin (0.50 g, resin loading, 100-200 mesh; 1.6 mmol g^-1^) was loaded into a 10 mL sinter-fitted syringe. Swelling of the resin took place in anhydrous DCM (4 ml) for 1 h. Afterwards, the resin was drained and a solution of Fmoc-L-Gly-OH (2 eq., 475.68 mg, 1.6 mmol, 297.3 g mol^-1^) and DIPEA (4 eq./amino acid, 1.088 mL, 6.4 mmol, 129.25 g mol-1, 0.76 g mL^-1^) in DCM (2 mL) was added to the resin. The resin was agitated at 500 rpm for 4 hours at room temperature. The resin was drained again and then washed with a mixture of DCM / DIPEA / methanol (17:1:2, 3 x 4 mL) to protect unreacted sites on the resin. After draining the resin was washed with DCM (3 x 4 mL), DMF (3 x 4 mL), DCM (3 x 4 mL) and DMF (3 x 4 mL). The resin, submerged in DMF (4 ml) was stored in the freezer (-20 °C) overnight. The removal of the Fmoc groups was carried out by the addition of 20 vol% piperidine in DMF (2 x 10 mL; 5 min each). A washing step after the removal of the deprotecting solution with DMF (3 x 4 mL), DCM (3 x 4 mL) and DMF (3 x 4 mL) was carried out. Subsequent coupling reactions were performed by adding solutions containing the Fmoc-L-Phe-OH (3 eq., 2.4 mmol, 929.76 mg, 387.40 g mol-1) or Fmoc-L-Gly-OH (3 eq., 2.4 mmol, 713.52 mg, 297.3 g mol-1), HBTU (3.1 eq., 2.48 mmol, 940.52 mg, 379.24 g mol-1) and DIPEA (6 eq., 4.8 mmol, 0.816 mL) in DMF (2 mL) to the resin. Coupling was proceeded at RT and agitating at 500 rpm for 3 hours. Deprotection and addition steps were sequentially repeated to obtain the desired tetrapeptide. Before and after each deprotection step, the resin was washed with DMF (3 x 4 mL), DCM (3 x 4 mL) and DMF (3 x 4 ml). After completion of the reaction sequence, the terminal Fmoc group was removed using 20 % piperidine in DMF. The peptide was cleaved from the resin using a solution of 20% HFIP in DCM (3 x 8 mL; 10 min each), where a colour change from yellow to red could be observed. The cleaved resin was washed with DCM (3 x 4 mL) and DMF (3 x 4 mL). The combined organic phases were removed under reduced pressure until ca. 2 ml were left, precipitated in DMF (5 mL), centrifuged and the supernatant removed. The white residue was washed with fresh DMF (2 mL) twice, transferred *via* MeOH into a glass vial and dried under reduced pressure to yield the product as a white powder.

^1^H-NMR (AcOH-d_4_, 300 MHz): *δ* = 2.85-3.25 (4H, m, -CH_2_-Phe), 3.87-4.12 (4H, m, -Gly), 4.82-4.94 (2H, dt, -CH-), 7.11-7.35 (10H, m, -Phe).

ESI-MS: [M + H]: 427.19, [M + Na^+^]: 449.18, [M + K^+^]: 465.15, [2M + H]: 853.38, [2M + Na^+^]: 875.36, [M + K^+^]: 891.34, [2M + Na^+^ + MeOH]: 907.30.

***General procedure for the copolymerization of BA and PDSA***

PABTC-NHS (24.2 mg, 73 μmol, 1 eq.), BA (841.5 mg, 6.6 mmol, 90.91 eq.), PDSA (158.5 mg, 0.7 mmol, 9.09 eq.) were weighed in a 20 mL microwave vial and charged with a magnetic stirrer. Afterwards, 1,4-dioxane (128 µL) and AIBN solution (30.6 µL of a 2 wt% solution in 1,4-dioxane, 3.65 μmol, 0.05 eq.) were added, the vial was sealed with a rubber septum and the solution degassed under stirring by a stream of nitrogen for 20 min. After degassing, a sample for ^1^H-NMR (50 µL, CDCl_3_) was taken and the vial suspended in a preheated oil bath at 70 °C for 48 h. The conversion (58 %) was determined by ^1^H-NMR (50 µL, CDCl_3_) by the ratio of the BA signal (-OCH_2_-) to the PBA signal (-OCH_2_-). Subsequently, the polymerization was quenched by cooling to room temperature and opening to air. The reaction mixture was dissolved in diethyl ether (1 mL), purified by precipitation in n-hexane (3 × 15 mL) and subsequent centrifugation (3 × 11000 rpm, 5 min) and decantation. The residue was dissolved in diethyl ether (2 mL), transferred in a screw cap vial and dried in a vacuum oven at 40 °C. The purified copolymer was analyzed by ^1^H-NMR (20 mg, CDCl_3_) and SEC (5 mg, DMAc + 0.21% LiCl).

^1^H-NMR (CDCl_3_, 300 MHz): *δ* = 0.93-0.97 (3H, t, *^3^J*, 6.0 Hz, BA, -CH_3_), 1.25-2.50 (7H, m, BA, -CH_2_-CH_2_-, backbone), 2.84 (0.07H, s, NHS), 3.05 (0.2H, s, PDSA, -CH_2_-S-S-), 4.05 (2H, s, BA, -O-CH_2_-), 4.33 (0.2H, s, PDSA, -O-CH_2_-), 7.12 (0.1H, s, PDSA, py), 7.71 (0.1H, s, PDSA, py), 8.49 (0.1H, s, PDSA, py).

SEC (eluent: DMAc + 0.21% LiCl, PMMA-standard): M_n_: 9 400 g mol^-1^, Ð = 1.09.

***Procedure for the polymer-polymer coupling***

P(BA-*co*-PDSA)-NHS (1.2 eq., 91 mg, 10.88 μmol) was dissolved in dry DMF (2 mL). mPEO-NH_2_ (1 eq., 50 mg, 9.06 μmol) was dissolved in dry DMF (2 mL) and triethylamine (2 eq., 2.51 μL) and added to the mixture at room temperature. The reaction mixture was stirred at room temperature for 24h. Subsequently, SEC (5 mg, DMAc + 0.21% LiCl, PMMA-calibration) was measured. After completion, DMF was evaporated, the polymer precipitated in Et2O/hexane 1/3, centrifuged (3 x 11 000, 5 min) and decanted to remove the excess of NHS polymer. Afterwards, the white precipitate was dissolved in acetone (5 mL) transferred into a screw cap vial, evaporated and dried under high vacuum overnight. SEC (5 mg, DMAc + 0.21% LiCl, PMMA-calibration) was measured after purification to determine the molar mass and dispersity.

^1^H-NMR (CDCl_3_, 300 MHz): *δ* = 0.91-0.95 (3H, t, *^3^J*, 6.0 Hz, BA, -CH_3_), 1.23-2.49 (7H, m, BA, -CH_2_-CH_2_-, backbone), 3.06 (0.2H, s, PDSA, -CH_2_-S-S-), 3.64 (9.49H, s, mPEO), 4.03 (2H, s, BA, -O-CH_2_-), 4.31 (0.2H, s, PDSA, -O-CH_2_-), 4.82-4.84 (0.037H, m, RAFT endgroup, -S-CH-CO-), 7.20 (0.1H, s, PDSA, py), 7.80 (0.1H, s, PDSA, py), 8.51 (0.1H, s, PDSA, py).

SEC (eluent: DMAc + 0.21% LiCl, PMMA-standard): M_n_: 17 700 g mol^-1^, Ð = 1.16.

***Procedure for the polymer-peptide coupling***

P(BA-*co*-PDSA)-NHS-ester (1.2 eq., 589.80 mg, 64 μmol) was dissolved in 2 ml DMF. Separately, Gly-Phe-Phe-Gly (GFFG) (1.0 eq., 22.78 mg, 53 μmol) and triethylamine (2.0 eq., 14.81 μL, 107 μmol) were dissolved in 2 ml DMF by gently heating due to the low solubility of the peptide, which gave a dispersion of the peptide. The P(BA-co-PDSA)-NHS solution was dropped into the peptide dispersion under stirring at room temperature. Subsequently, the combined mixture was stirred for 48 hours at a constant temperature. Afterwards, a clear yellow solution was obtained. Benzylamine functionalized scavenger resin was added in excess to the solution and stirred for 5 h to bind any residual P(BA-*co*-PDSA)-NHS-ester. Finally, the resin was filtered off and DMF and triethylamine were removed in vacuo. ^1^H-NMR (10 mg, AcOH-d_4_, 300 MHz) and SEC (5 mg, DMAc + 0.21 wt% LiCl, PMMA-calibration) was measured to analyze the obtained polymer-peptide.

^1^H-NMR (AcOH-d_4_, 300 MHz): *δ* = 1.00 (3H, s, BA, -CH_3_), 1.44-2.60 (7H, m, BA, -CH_2_-CH_2_-, backbone), 3.14 (0.27H, s, PDSA, -CH_2_-S-S-, GFFG, -CH_2_-Phe), 3.82-4.30 (2.07H, s, BA, -O-CH_2_-, GFFG, -Gly), 4.40 (0.2H, s, PDSA, -O-CH_2_-), 4.83 (0.017H, s, RAFT endgroup, -S-CH-CO-), 4.92 (0.034H, s, GFFG, -CH-), 7.10-7.40 (0.27H, m, PDSA, py, GFFG, -Phe), 7.94 (0.1H, m, PDSA, py), 8.60 (0.1H, s, PDSA, py).

SEC (eluent: DMAc + 0.21% LiCl, PMMA-standard): M_n_: 11 800 g mol^-1^, Ð = 1.18.

***Procedure for the polymer-peptide-polymer coupling***

P(BA-*co*-PDSA)-(GFFG) (1 eq., 53.43 mg, 5.62 μmol), 1-Ethyl-3-(3-dimethylaminopropyl)carbodiimide hydrochloride (EDC, 5 eq., 5.39 mg, 28 μmol) and 4-(Dimethylamino)-pyridin (DMAP, 0.5 eq., 69 μg, 0.56 μmol) were dissolved in dry DMF (2 mL). ɑ-methoxy-ω-amino-PEO (1.5 eq., 46.5 mg, 8.44 μmol) was dissolved separately in dry DMF (2 mL). For the complete dissolution of PEO, the solution was heated gently. The solutions were combined by dropping the PEO-solution via a syringe pump (2 mL h^-1^) into the PBA-peptide solution while stirring at RT. Addition with a syringe pump was chosen to keep the concentration of any unreacted amino-functional PEO low minimizing undesired side reaction with the trithiocarbonate end group. After stirring overnight at RT, the successful coupling reaction was tested by SEC (5 mg, DMAc + 0.21% LiCl, PMMA-calibration). Subsequently, the DMF was evaporated in vacuo at 40 °C. Deionized water (15 mL) was added to the residue and suspended by shaking and heating. The dispersion was transferred into an amicon ultracentrifuge tube (MWCO: 10 kDa). Therefore, the product was isolated by 10 centrifugation runs at 8000 rpm for 30 min. The residue was dissolved in acetone (5 mL) transferred into a screw cap vial, evaporated and dried under high vacuum overnight. SEC (5 mg, DMAc + 0.21% L iCl, PMMA-calibration) was measured after purification.

^1^H-NMR (AcOH-d_4_, 300 MHz): *δ* = 1.00 (3H, s, BA, -CH_3_), 1.41-2.70 (7H, m, BA, -CH_2_-CH_2_-, backbone), 3.14 (0.27H, s, PDSA, -CH_2_-S-S-, GFFG, -CH_2_-Phe), 3.66-3.76 (9.49H, mPEO),

4.13 (2.07H, s, BA, -O-CH_2_-, GFFG, -Gly), 4.40 (0.2H, s, PDSA, -O-CH_2_-), 7.23-7.32 (0.27H, m, PDSA, py, GFFG, -Phe), 7.94 (0.1H, m, PDSA, py), 8.60 (0.1H, s, PDSA, py).

SEC (eluent: DMAc + 0.21% LiCl, PMMA-standard): M_n_: 19 300 g mol^-1^, Ð = 1.19.

Micelle formulation

The block copolymer (peptide) (10 mg) was dissolved in DMSO (2.50 mL) and ultrapure water (2.50 mL) was added successively *via* syringe pump (1 mL h^-1^) under vigorously stirring. Afterwards, the mixture was dialyzed for 1 day (2 solvent exchanges, MWCO: 3.5 kDa) against deionized water to remove the cosolvent. The successful micelle formation was monitored by DLS. In the next step, the core-crosslinking was induced by the addition of 1,6-hexanedithiol solution (363 µL, 1 mg mL^-1^ in DMSO, 0.5 eq. of PDSA groups) and stirred overnight at room temperature. Subsequently, the mixture was dialyzed against deionized water for 4 days (5 water exchanges, MWCO: 3.5 kDa) to remove the cosolvent and any residual crosslinker. Afterwards DLS was measured and the final concentration of the micelle dispersion determined gravimetrically (*n* = 3) after lyophilization.

***Cryo-TEM investigations***

The measurements were performed on a FEI Tecnai G² 20 platform with a LaB6 filament at 200 kV acceleration voltage. Samples were prepared on carbon coated TEM grids (Plano) or Quantifoil grids (R2/2) which were both treated with Ar plasma prior to use for hydrophilization and cleaning. 15 µL of the solutions were blotted onto the carbon coated films for TEM investigation, while for cryogenic transmission electron microscopy (cryo-TEM) measurements 8.5 µL of the solution were vitrified on Quantifoil grids using a Vitrobot Mark IV system. Liquid ethane was used as a cryogen. Samples were transferred to a Gatan 626 cryo holder and were maintained at a temperature < ‑175 °C during the entire process. All images were acquired with a Mega View (OSIS, Olympus Soft Imaging Systems) or an Eagle 4k CCD camera, respectively.

***Atomic force microsocopy***

The samples were characterized using a NanoWizard III Bioscience AFM system (Bruker, Germany). Topography and phase images were acquired in intermittent mode employing commercial silicon cantilevers CSG30 **(Spectrum Instruments Ltd.).** The probes have a typical resonance frequency of 48 kHz and a force constant of 0.6 N/m. Data acquisition was managed with SPM Control Software v.6 (Bruker, Germany). Large-scale topography images, utilized to determine the contour and persistence lengths of the filomicelles, were captured at a resolution of 1024×1024 pixels or higher to enable resolving fine structural details. Post-processing of AFM images was performed using Gwyddion 2.53 software.[[3](#_ENREF_3)] Standard plane and line flattening procedures were applied to correct topography and phase images. Nanomechanical characterization of the samples was performed using JPK's Quantitative Imaging (QI™) mode, employing the same type of cantilever. Adhesion maps were generated from the acquired force-distance curves and are presented as raw data.

***Tip-enhanced Raman scattering***

TERS spectra were recorded using the same AFM setup to precisely control the tip-sample distance. A Raman spectrometer was integrated with the SPM platform, built on an inverted microscope (IX71, Olympus, Germany). The microscope featured a 60× oil immersion objective (NA 1.45). A linearly polarized laser beam with a 532 nm excitation wavelength was focused on the tip-sample region via the objective, with an excitation power of ~ 600 µW at the sample. The Raman signal was collected using the same objective and separated from the excitation light through a dichroic beamsplitter (razor edge 532, Semrock Inc., USA) and an edge filter (532 US LPF, Iridian, Canada). The signal was then directed to a spectrograph (Acton Advanced SP 2750A, Princeton Instruments, USA) equipped with a Peltier-cooled CCD camera (Pixis 256, Princeton Instruments, USA). Each spectrum was acquired with an exposure time of 1–2 s.

The TERS tips consisted of commercial silicon cantilevers (Tap 190-Al, Budget Sensor, Bulgaria) decorated with Ag nanoparticles. These nanoparticles were fabricated by evaporating a 2 nm-thick Ag film onto the cantilever tips, followed by annealing, which produced individual Ag nanoparticles at the tip apex. To prevent oxidation, the Ag-decorated tips were stored under an argon atmosphere until use. This type of Ag nanoparticle tips are characterized by a high sensitivity, signal stability and reproducibility (see also Figure S6 and S7). [[4-8](#_ENREF_4)]

**Data analysis**

***TERS spectra***

Raw TERS spectra were processed to remove cosmic rays by omitting the affected pixels. For enhanced visualization, spectra presented in waterfall plots underwent baseline correction using asymmetric least squares (AsLS) fitting. To facilitate band deconvolution in the amide spectral regions and for the F1-mode of the phenylalanine residue, AsLS baseline subtraction was applied, followed by gentle smoothing using the Savitzky–Golay method. Band positions were determined through multiple peak fitting employing Lorentzian functions. The average TERS spectra of Figure 3a comprise 200 and 500 individual TERS spectra respectively, with an integration time of 1s recorded at different step sizes (min. 1 nm) along individual filomicelles. In case of the PBA-b-(GFFG)-b-PEO filomicelles, a line averaging across two adjacent, tightly connected filomicelles was applied, to map across a statistical meaningful sampling area,[[9](#_ENREF_9)] which accounts for the higher chemical heterogeneity of these filomicelles due to the peptide interfacial shell.

***Determination of the contour length, persistence length, bending rigidity and elastic modulus.***

The contour length, persistence length, bending rigidity, and elastic modulus of the filomicelles were determined using the open-source software tool ***Easyworm***, specifically designed for analyzing the nanomechanical properties of individual worm-like chain biopolymers.[[10](#_ENREF_10)] Large-scale, high-resolution AFM topography images containing a sufficient number of filomicelles, including extremely long ones (>5 µm), were analyzed. Initially, the contours of the filomicelles were fitted to parametric splines, yielding the contour profiles and lengths.

The persistence length was derived based on the worm-like chain model for semi-flexible polymers, using two approaches: tangent-tangent correlation analysis and mean square end-to-end distance measurements, as detailed in Ref.[[10](#_ENREF_10)] and SIs. Both methods provided consistent values for the persistence length. These results enabled the calculation of the axial elastic modulus *E* of the filomicelles using the relationship $E=P k_{B}T/I$, where *T* is the temperature, *k_B_*​ is the Boltzmann constant, and I is the cross-sectional second moment of area.

To approximate I, a circular cross-section was assumed, calculated as $I=\pi h^{4}/64$, where *h* represents the average height of the filomicelles observed in the AFM images.[[10](#_ENREF_10)] Height data were extracted by masking the centers of the filomicelles. To account for underestimation of the real height caused by drying artifacts, an empirical correction factor of ^3^/_2_ was applied.

**Characterisation of polymers**

**Table S1.** Overview of the synthesized polymers and assembled micelles including abbreviations and characterization.

| ID | Polymer | M_n,theo_^a)^  [g mol^-1^] | M_n,SEC_^b)^  [g mol^-1^] | Ð^b)^ | D_h_ ^c)^  [nm] | PDI ^c)^ |
| --- | --- | --- | --- | --- | --- | --- |
| P1 | P(BA_54.1_-*co*-PDSA_5.4_)-NHS | 8 600 | 9 400 | 1.09 | - | - |
| P2 | P(BA_58.0_-*co*-PDSA_5.8_)-NHS | 9 200 | 9 900 | 1.14 | - | - |
| P3 | P(BA_54.1_-*co*-PDSA_5.4_)-*b*-PEO_125_ | 14 000 | 17 700 | 1.16 | - | - |
| P4 | P(BA_58.0_-*co*-PDSA_5.8_)-*b*-(GFFG) | 9 500 | 11 800 | 1.18 | - | - |
| P5 | P(BA_58.0_-*co*-PDSA_5.8_)-*b*-(GFFG)-*b*-PEO_125_ | 15 000 | 19 300 | 1.19 | - | - |
| M1 | P(BA_54.1_-*co*-PDSA_5.4_)-*b*-PEO_125_ | - | - | - | 107.4 ± 0.8 | 0.19 ± 0.00 |
| M2 | P(BA_58.0_-*co*-PDSA_5.8_)-*b*-(GFFG)-*b*-PEO_125_ | - | - | - | 157.3 ± 2.9 | 0.11 ± 0.03 |

a) Calculated based on [M]_0_/[CTA]_0_ × monomer conversion. B) Determined by SEC (Eluent: DMAc + 0.21 wt% LiCl, PMMA-calibration). c) Determined by DLS measurements of the purified self-assembled structures (three technical replicates, c: 1 mg mL^-1^).

**(B)**

**(A)**


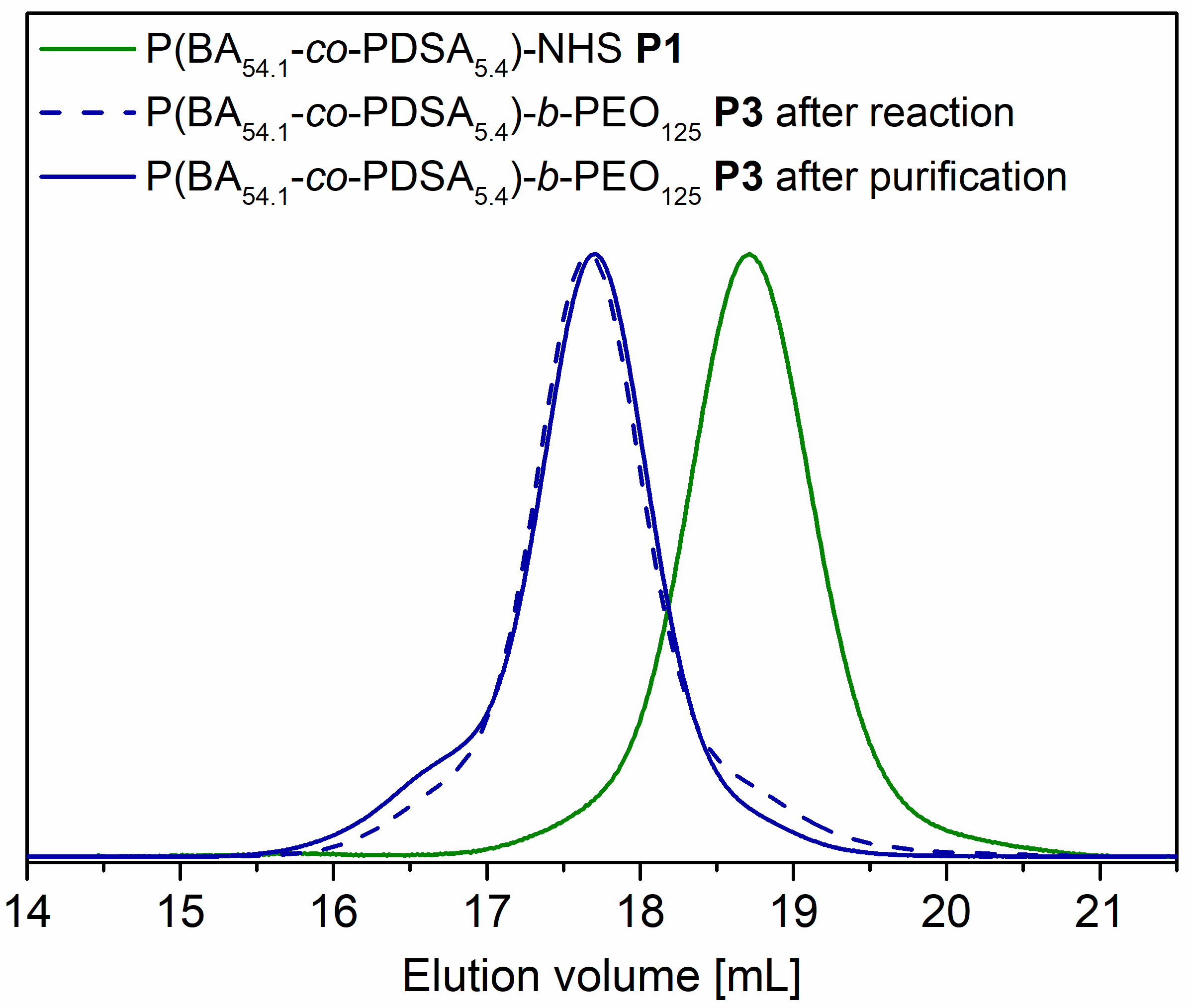

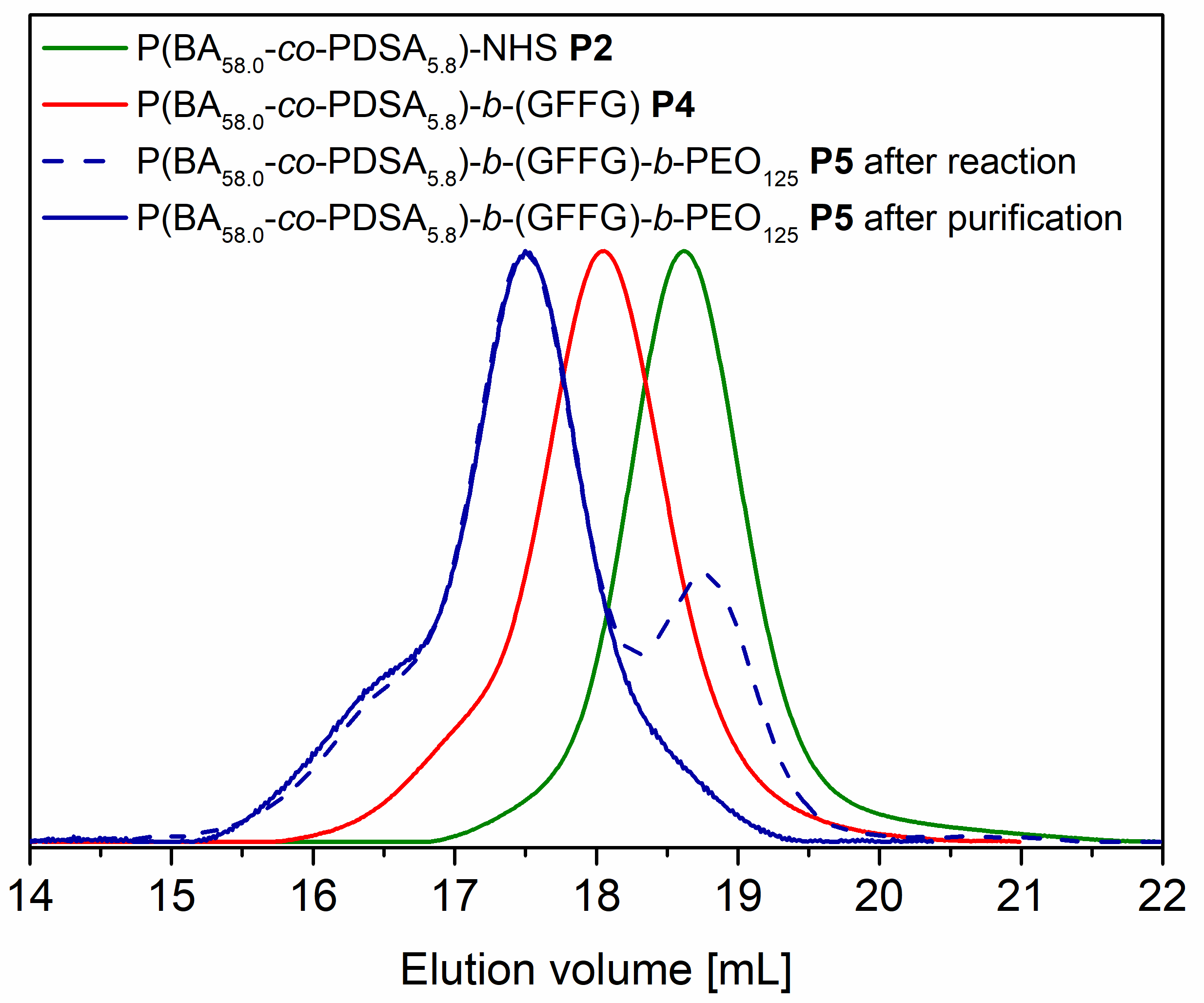


**Figure S1.** A) Overlay of the SEC curves of the samples **P1** and **P3** (control polymer). (Eluent: DMAc + 0.21% LiCl, PMMA-calibration). B) Overlay of the SEC curves of the samples **P2**, **P4** and **P5** (peptide containing polymer). (Eluent: DMAc + 0.21% LiCl, PMMA-calibration).


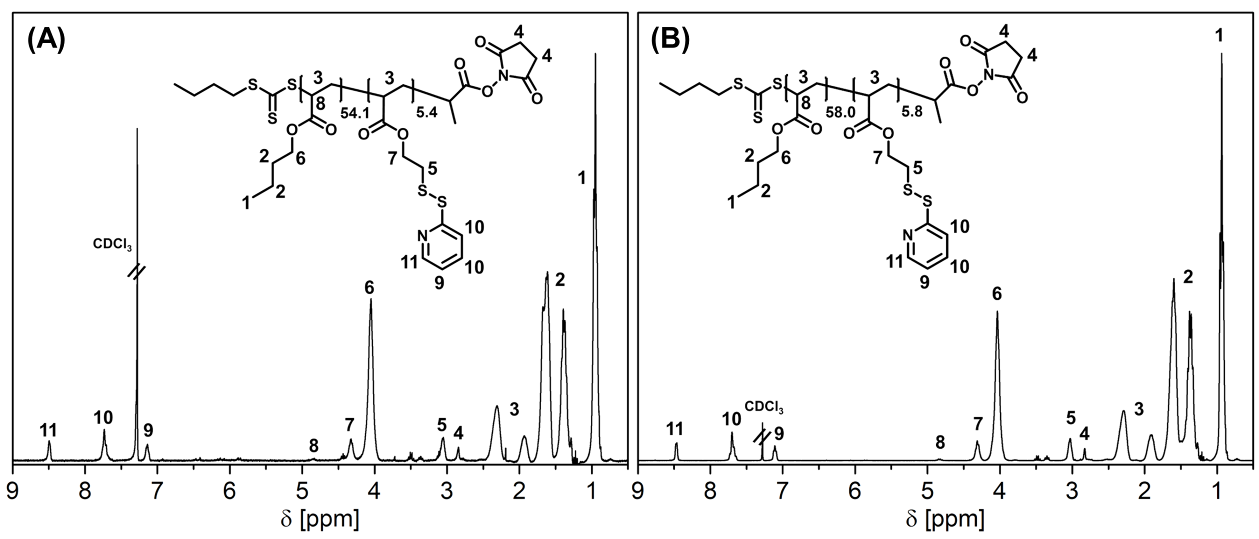


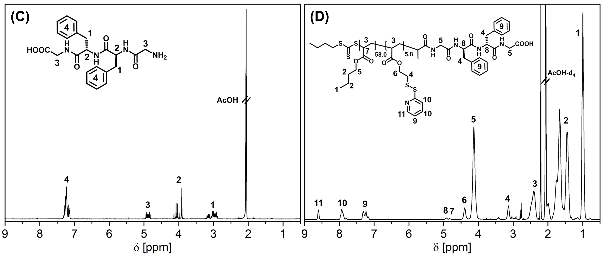


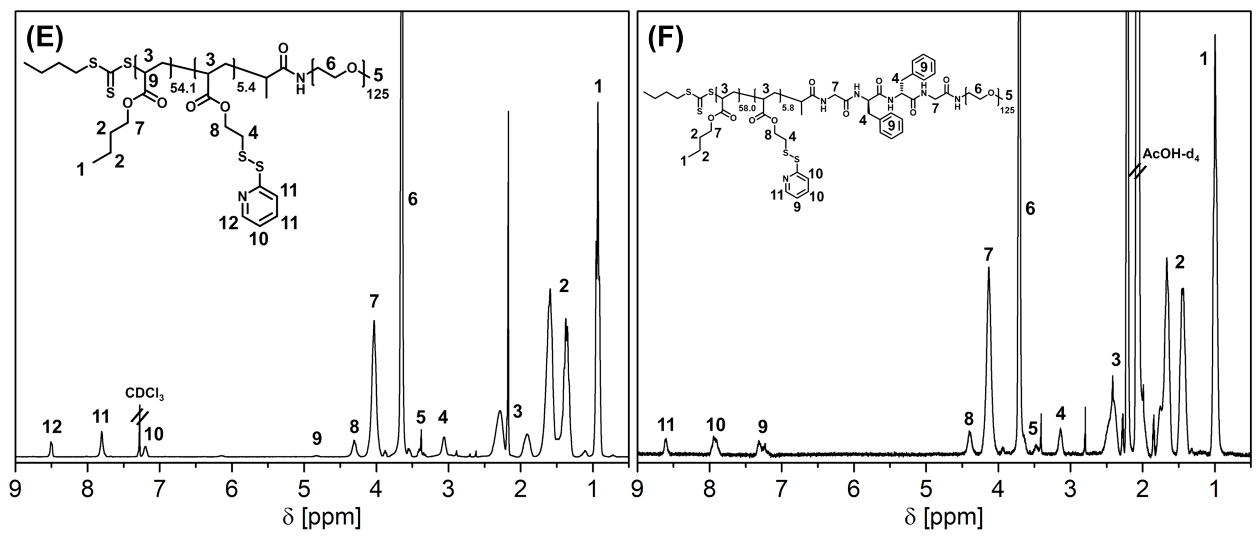


**Figure S2.** Overview of the ^1^H-NMR (300 MHz) spectra measured for all samples after purification. A) P(BA_54.1_-*co*-PDSA_5.4_)-NHS **P1** in CDCl_3_. B) P(BA_58.0_-*co*-PDSA_5.8_)-NHS **P2** in CDCl_3_. C) GFFG in AcOH-d_4_. D) P(BA_58.0_-*co*-PDSA_5.8_)-*b*-(GFFG) **P4** in AcOH-d_4_. E) P(BA_54.1_-*co*-PDSA_5.4_)-*b*-mPEO_125_ **P3** in CDCl_3_. F) P(BA_58.0_-*co*-PDSA_5.8_)-*b*-(GFFG)-*b*-mPEO_125_ **P5** in AcOH-d_4_.


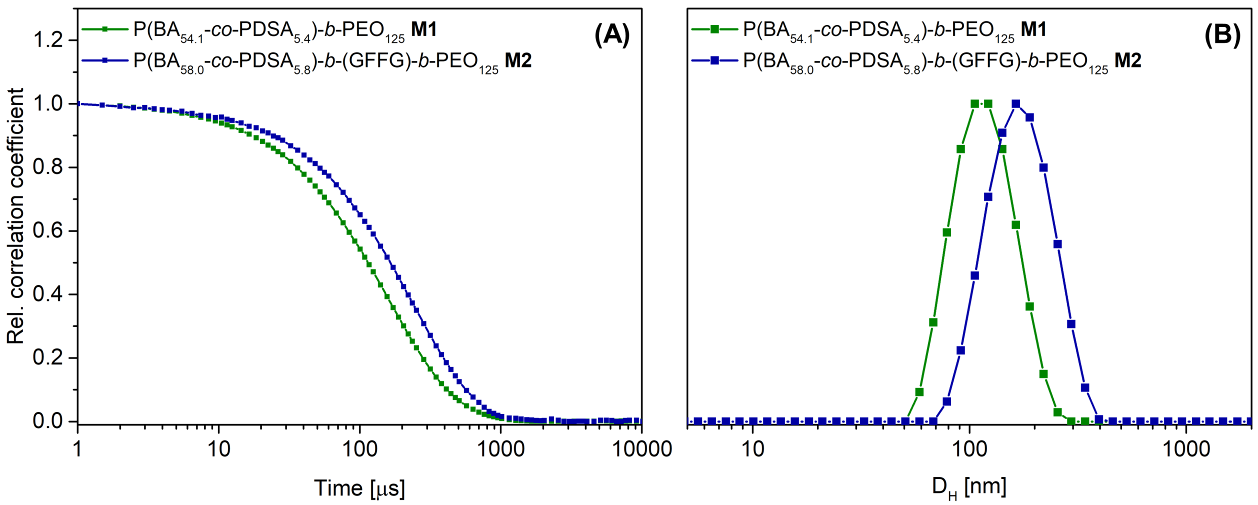


**Figure S3.** DLS analysis of filomicelles **M1** and **M2** (three technical replicates, c: 1 mg mL^-1^). A) Correlation functions of **M1** and **M2**. B) Intensity-averaged size distribution of **M1** and **M2**.

**Analysis of self-assembled structures**


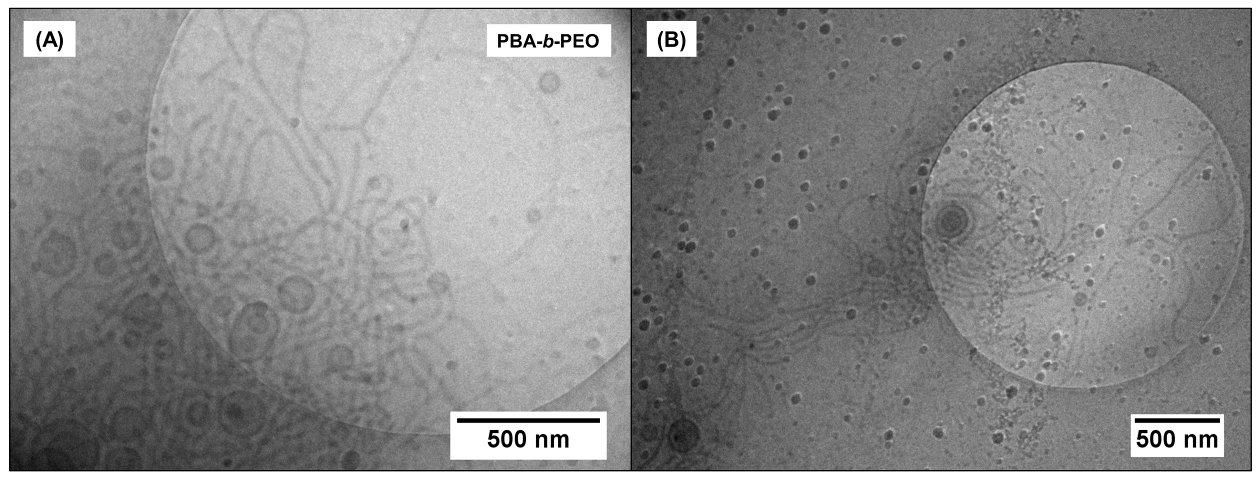

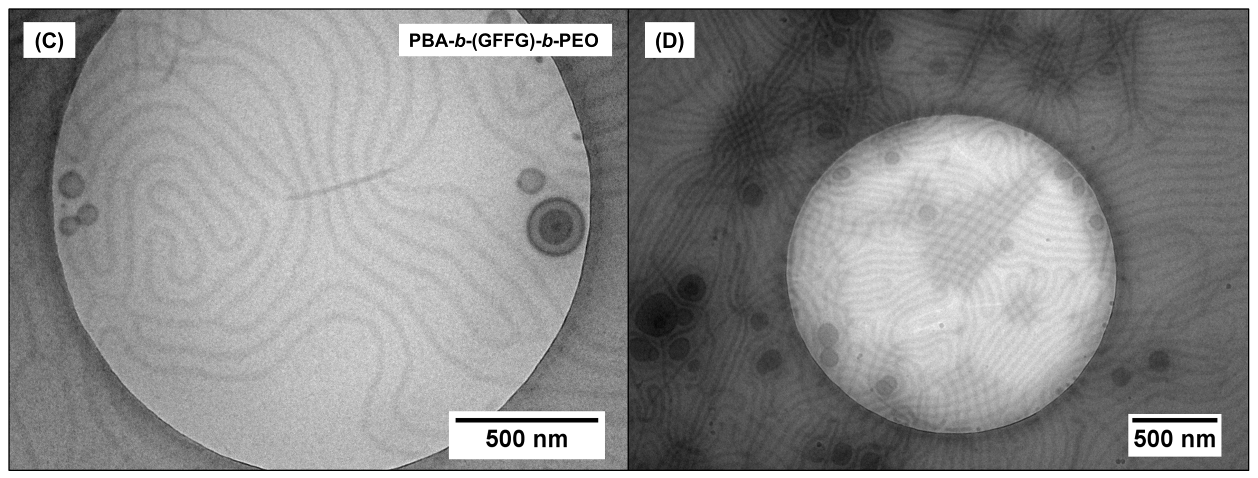


**Figure S4.** Additional Cryo-TEM images with different magnifications of filomicelles **M1** (A, B) and **M2** (C, D).


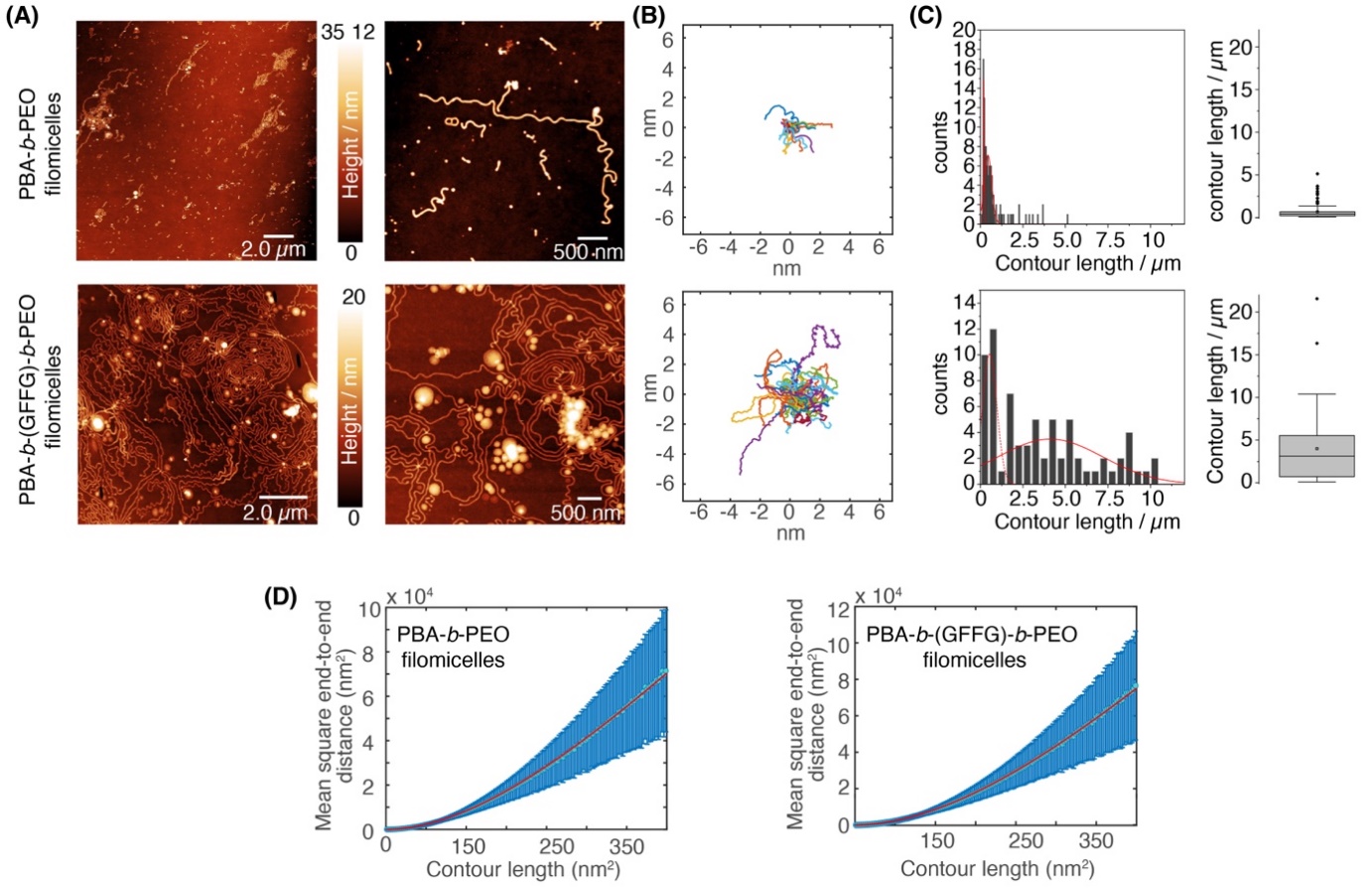


**Figure S5.**  (A) High resolution AFM overview images of the block copolymer (PBA-*b*-PEO) (bottom) and (PBA-*b*-(GFFG)-*b*-PEO) (top) filomicelles. (B) Representation of the bending affinity and contour length distribution. (C) Extraction of the persistence length by application of a wormlike chain model. (D) Calculation of the persistence length by measuring the mean square of the end-to-end distance. Red lines correspond to the fits of the worm-like chain model to the data.

**Table S2** Comparison of characteristic geometrical and mechanical parameters for both filomicelles

| **Parameter** | **PBA-*b*-(GFFG)-*b*-PEO** | **PBA- *b*-PEO** |
| --- | --- | --- |
| **Height AFM** | 7 (± 1 nm) | 5 (± 1 nm) |
| **Diameter TEM** | 27.4 (± 3.1 nm) | 25.1 (± 2.9 nm) |
| **Average Contour Length** ^(*)^ | 3998 (±3 916 nm) | 694 (± 845 nm) |
| **Persistence Length** | 152 (± 53 nm) | 132 (± 46 nm) |
| **Bending Rigidity** | 6.3 10^-28^ (± 2.2 10^-28^ Nm^2^) | 5.4 10^-28^ (± 1.9 10^-28^ Nm^2^) |
| **Elastic Modulus**  **AFM**  Meas. height 7 nm/5 nm  Corr. Height 11 nm / 8 nm | 5.3 (±2.4 MPa)  0.9 (±0.6 MPa) | 16,4 (±6.1 MPa)  2.7 (±1.4 MPa) |

^(*)^ Both values exhibit a high standard deviation, which is caused by considering only 74 and 119 filomicelles respectively. As reflected by the histograms in Figure S6 (c) the large spread in the contour length over more than one order of magnitude would requires a larger number of samples, which is rather limited by the number of filomicelles per image.

**Table S3** Raman band assignment

|  | **Assignment*** | **Wavenumber / cm^-1^** |
| --- | --- | --- |
| **Peptide Shell** | **Amide I**  **Amide II**  **Amide III** | 1700-1616  1560-1520  1300-1220 |
| **Phenyl alanine** | F_1_  F_2_  F_3_  F_4_  F_5_  F_6_ | 1606  1586  1207  1032  1004  622 |
| **PEO corona** | γ(CH_2)_ +ν(CO) trans  γ(CH_2)_ +ν(CO) gauche  ν(CO) trans  ν(CO) gauche | 820  880  1083  1124 |
| **PBA/PSDA core** | ν(C=O)  ν(CS)  ν(SS) | 1780-1720  630-650  490-530 |

(*) Band assignment of the amide bands and structural features follows Ref. [[11-13](#_ENREF_11)] and references therein. Band assignment of the phenylalanine Fi-modes according to Ref. [[14](#_ENREF_14)]. PEO band assignment according to Ref. [[15](#_ENREF_15), [16](#_ENREF_16)] and PBA/PSDA assignment according to Ref. [[17](#_ENREF_17), [18](#_ENREF_18)].

Individual TERS spectra recorded on a **PBA- *b*-PEO filomicelle**


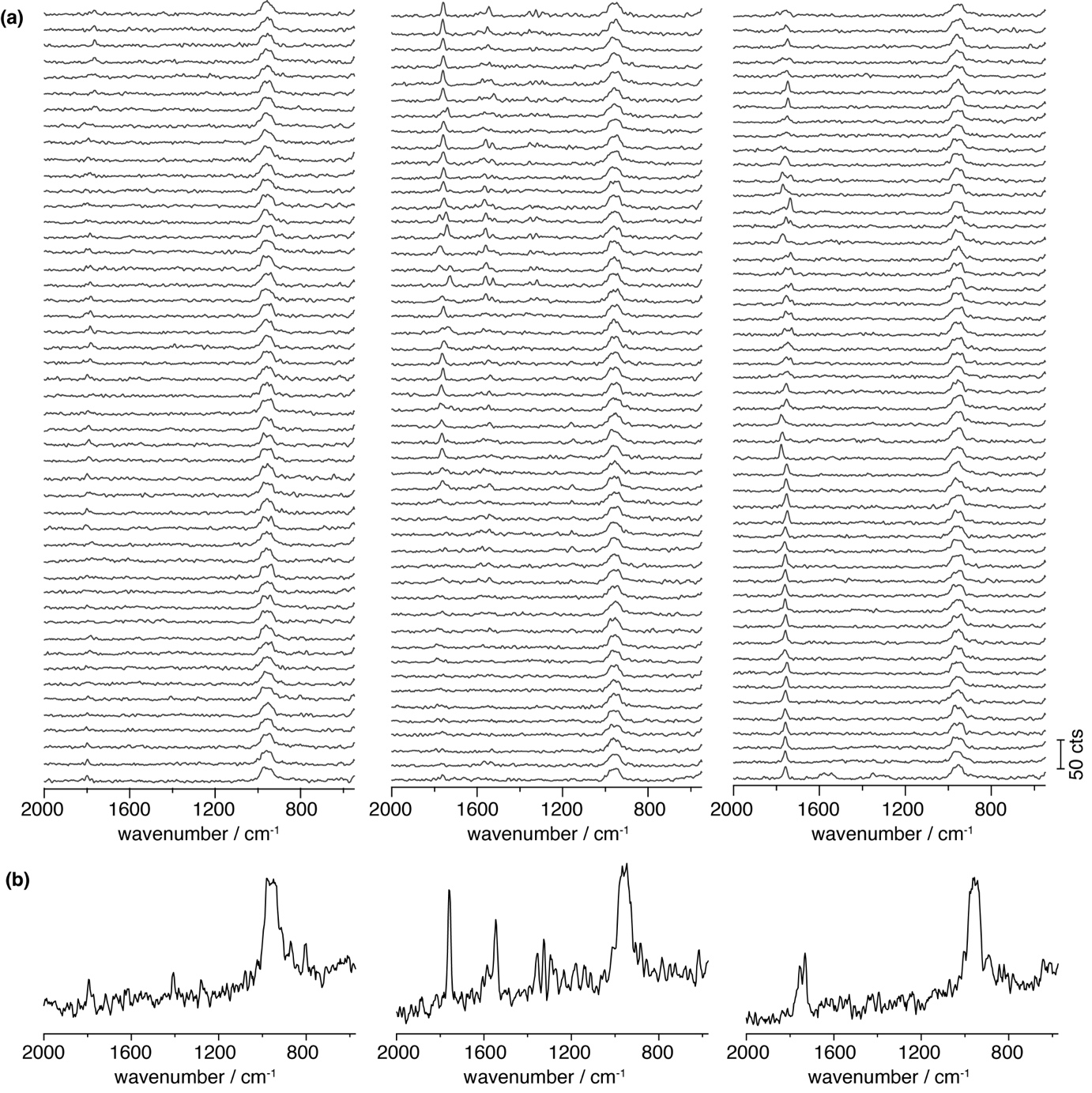


**Figure S6.** (a) 150 individual TERS spectra averaged in Figure 3 demonstrating the high reproducibility of the TERS investigations. (b) Individual TERS spectra representing the local spectral variations, according to different contributions of the PEO shell and the crosslinked P(BA-b-PDSA) core.

Individual TERS spectra recorded on **PBA-*b*-(GFFG)-*b*-PEO filomicelles**


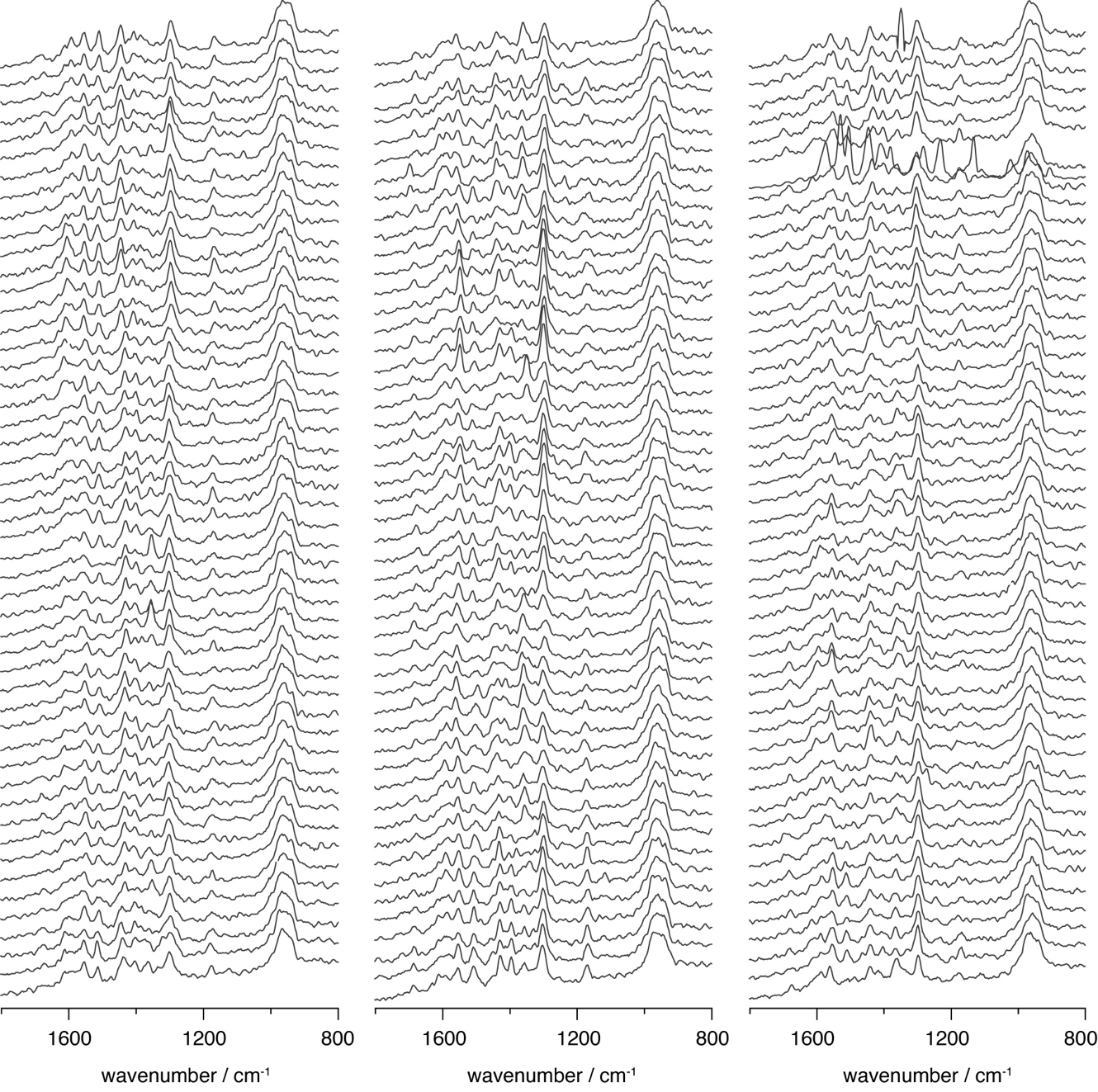


**Figure S7.** 150 out of 500 individual TERS spectra averaged in Figure 3 recorded on **PBA-*b*-(GFFG)-*b*-PEO filomicelles.** A high reproducibility of the TERS investigations is observed, despite the higher chemical heterogeneity introduced by the peptide shell.

­­
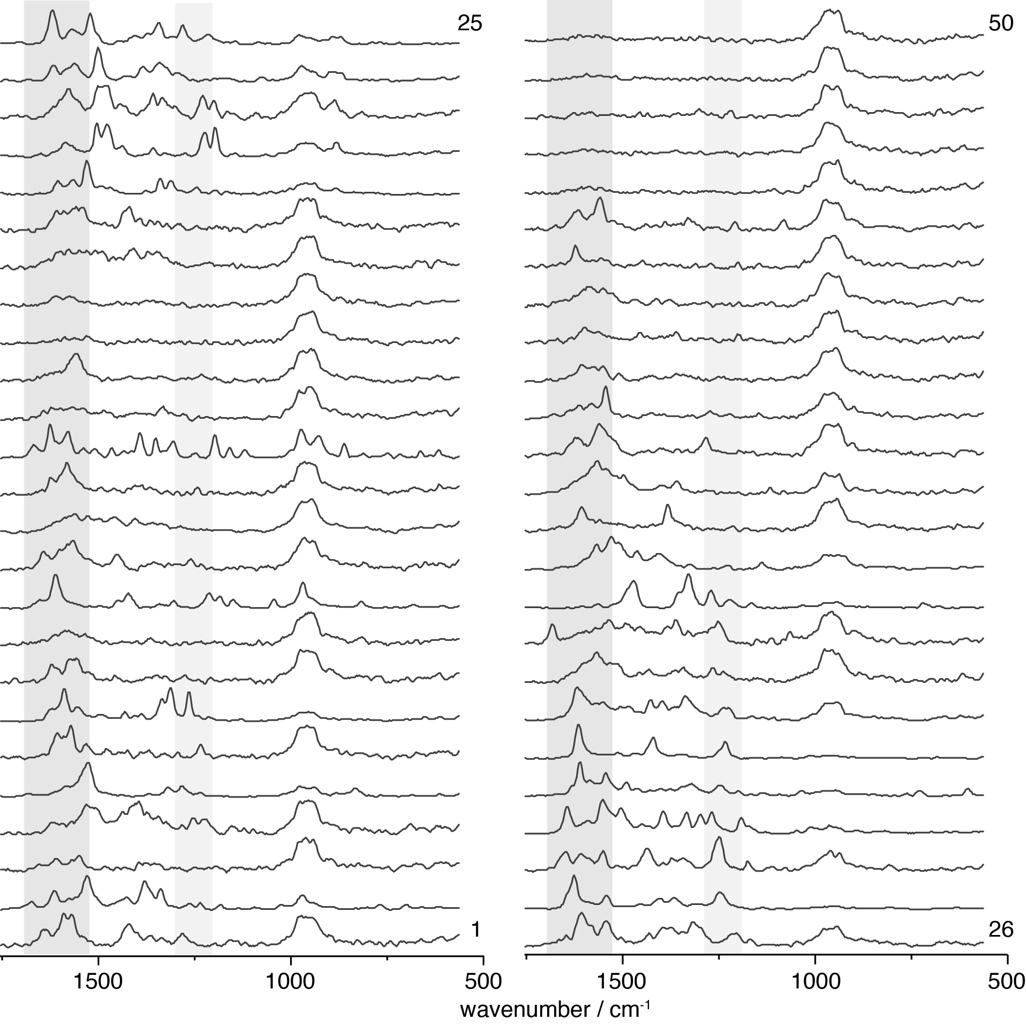


**Figure S8.** TERS spectra of Figure 4 displaying a spectral region form 500-1650 cm^-1^. Grey and light grey boxes mark the amide I / II and amide III region. All spectra are shown in a sequential order (left: TERS spectrum numbers 1 to 25 and right TERS spectra number 26-50, which correspond to the position in Figure 4).

TERS mapping of **PBA-*b*-(GFFG)-*b*-PEO filomicelles**


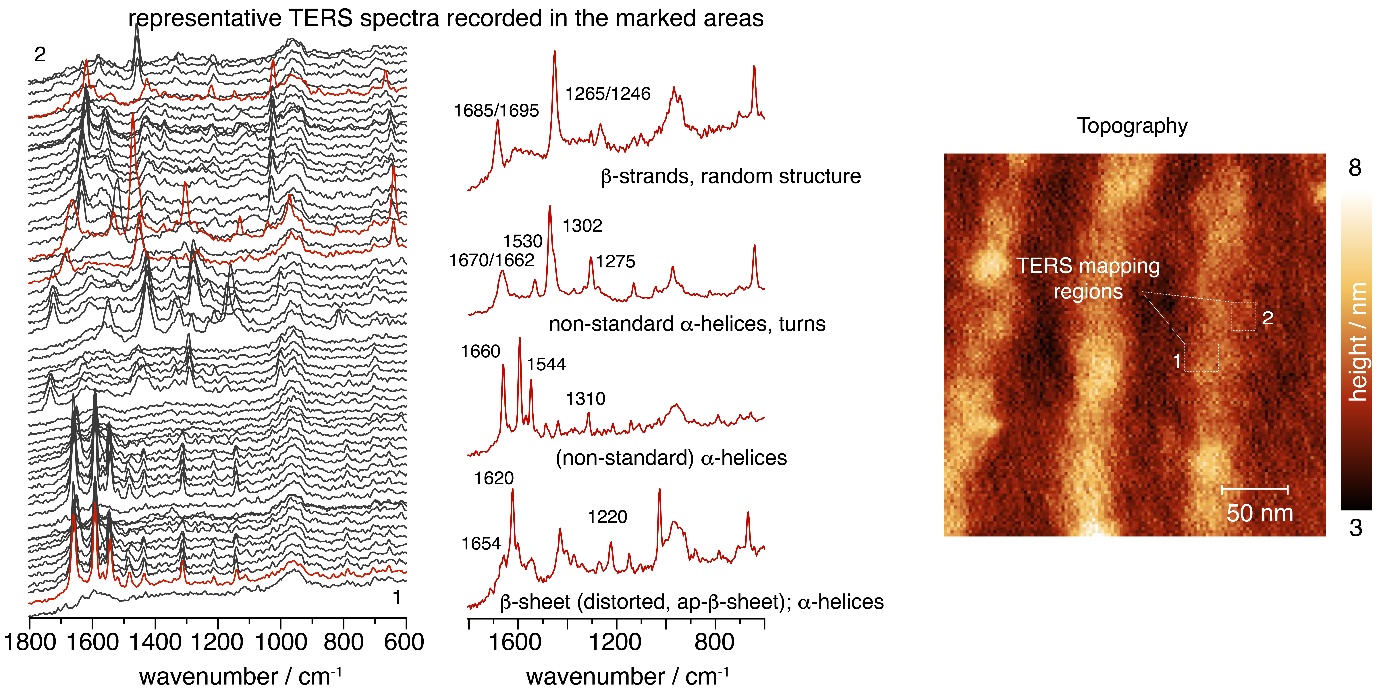


**Figure S9.** TERS mapping of the marked regions in the topography image of PBA-*b*-(GFFG)-*b*-PEO filomicelles. The individual TERS spectra confirm the larger chemical and structural heterogeneity imposed by the introduced peptide interface.

**References**

1. K. C, R. B.; Thapa, B.; Xu, P., *Mol. Pharmaceutics* **2012,** *9* (9), 2719-2729. DOI 10.1021/mp300274g.

2. Larnaudie, S. C.; Brendel, J. C.; Jolliffe, K. A.; Perrier, S., *J. Polym. Sci., Part A: Polym. Chem.* **2016,** *54* (7), 1003-1011. DOI 10.1002/pola.27937.

3. Nečas, D.; Klapetek, P., *Open Phys.* **2012,** *10* (1), 181-188. DOI doi:10.2478/s11534-011-0096-2.

4. Bailo, E.; Deckert, V., *Angew. Chem. Int. Ed.* **2008,** *47* (9), 1658-1661. DOI 10.1002/anie.200704054.

5. Deckert-Gaudig, T.; Bailo, E.; Deckert, V., *Phys. Chem. Chem. Phys.* **2009,** *11* (34), 7360-7362. DOI 10.1039/B904735B.

6. Deckert-Gaudig, T.; Rauls, E.; Deckert, V., *J. Phys. Chem. C* **2010,** *114* (16), 7412-7420. DOI 10.1021/jp9098045.

7. Richard-Lacroix, M.; Deckert, V., *Light: Science & Applications* **2020,** *9* (1), 35. DOI 10.1038/s41377-020-0260-9.

8. Richard-Lacroix, M.; Küllmer, M.; Gaus, A.-L.; Neumann, C.; Tontsch, C.; von Delius, M.; Deckert, V.; Turchanin, A., *Advanced Materials Interfaces* **2022,** *9* (14), 2102389. DOI 10.1002/admi.202102389.

9. Höppener, C.; Aizpurua, J.; Chen, H.; Gräfe, S.; Jorio, A.; Kupfer, S.; Zhang, Z.; Deckert, V., *Nature Reviews Methods Primers* **2024,** *4* (1), 47. DOI 10.1038/s43586-024-00323-5.

10. Lamour, G.; Kirkegaard, J. B.; Li, H.; Knowles, T. P. J.; Gsponer, J., *Source Code Biol. Med.* **2014,** *9* (1), 16. DOI 10.1186/1751-0473-9-16.

11. Lipiec, E.; Kaderli, J.; Kobierski, J.; Riek, R.; Skirlińska-Nosek, K.; Sofińska, K.; Szymoński, M.; Zenobi, R., *Angew. Chem. Int. Ed.* **2021,** *60* (9), 4545-4550. DOI <https://doi.org/10.1002/anie.202010331>.

12. Kuhar, N.; Sil, S.; Umapathy, S., *Spectrochimica Acta Part A: Molecular and Biomolecular Spectroscopy* **2021,** *258*, 119712. DOI <https://doi.org/10.1016/j.saa.2021.119712>.

13. Barth, A.; Zscherp, C., *Q. Rev. Biophys.* **2002,** *35* (4), 369-430. DOI 10.1017/S0033583502003815.

14. Hernández, B.; Pflüger, F.; Kruglik, S. G.; Ghomi, M., *J. Raman Spectrosc.* **2013,** *44* (6), 827-833. DOI <https://doi.org/10.1002/jrs.4290>.

15. Höppener, C.; Elter, J. K.; Schacher, F. H.; Deckert, V., *Small* **2023,** *19* (20), 2206451. DOI <https://doi.org/10.1002/smll.202206451>.

16. Samuel, A. Z.; Umapathy, S., *Polym. J.* **2014,** *46* (6), 330-336. DOI 10.1038/pj.2014.10.

17. Hernández, B.; Pflüger, F.; López-Tobar, E.; Kruglik, S. G.; Garcia-Ramos, J. V.; Sanchez-Cortes, S.; Ghomi, M., *J. Raman Spectrosc.* **2014,** *45* (8), 657-664. DOI <https://doi.org/10.1002/jrs.4521>.

18. Socrates, G., *Infrared and Raman Characteristic Group Frequencies: Tables and Charts*. 3rd ed.; John Wiley & Sons: **2004**.
